# Bibliometric Analysis of the English Musculoskeletal Literature over the Last 30 Years

**DOI:** 10.1155/2021/5548481

**Published:** 2021-04-27

**Authors:** Randall T. Loder, Melissa A. Kacena, Blessing Ogbemudia, Hervé Nonga Ngwe, Abdul Aasar, Nehal Ninad, Osama Mufti, Zachary Gunderson, Elizabeth C. Whipple

**Affiliations:** ^1^James Whitcomb Riley Children's Hospital, Indianapolis, IN, USA; ^2^Department of Orthopaedic Surgery, Indiana University School of Medicine, Indianapolis, IN, USA; ^3^University of Florida, Department of Anesthesiology, Gainesville, FL, USA; ^4^Indiana University School of Medicine, Student Affairs, Indianapolis, IN, USA; ^5^Department of Radiology, Indiana University School of Medicine, Indianapolis, IN, USA; ^6^Department of Surgery, Mercer University School of Medicine, Macon, GA, USA; ^7^Department of Ophthalmology, SUNY Downstate Medical Center, Brooklyn, NY, USA; ^8^Ruth Lilly Medical Library, Indiana University School of Medicine, Indianapolis, IN, USA

## Abstract

Publication and authorship are important in academia for career advancement, obtaining grants, and improved patient care. There has been a recent interest in bibliometric changes over time, especially regarding the gender gap. The purpose of this study was to explore bibliometric changes in the musculoskeletal literature. Bibliometric variables (number of authors, institutions, countries, pages, references, corresponding author position, author gender, geographic region of origin, and editorial board makeup) were analyzed for 5 basic science and 12 clinically oriented musculoskeletal journals from 1985 through 2016. Statistical analyses comprised bivariate analyses, multifactorial ANOVAs, and logistic regression analyses. A *p* < 0.005 was considered significant. Nearly, all variables increased over time. Asia had the highest number of authors and corresponding author positions, Australia/New Zealand the highest number of institutions and references, North America the highest number of pages, and Europe the highest number of countries. Those with a female first author had more authors, institutions, countries, references, and pages. Likewise, those with a female corresponding author had more authors, institutions, countries, references, and pages. Single-authored manuscripts decreased over time. The percentage of female first authors rose from 10.8% in 1985–1987 to 23.7% in 2015–2016. There were more female 1^st^ authors in the basic science journals compared to the clinical journals (33.2% vs. 12.7%). Single-authored manuscripts were more likely to be written by males (5.1 vs. 2.4%) and decreased over time. The many differences by geographic region of origin likely reflect different socio/cultural attitudes regarding academia and research, as well as the gender composition of the disciplines by geographic region. Overall, there has been an increase in the number of female 1^st^ and corresponding authors, editorial board members, and chief editors, indicating a slow but progressive narrowing of the gender gap.

## 1. Introduction

There has been a recent interest in studying bibliometric changes overtime for (1) the scientific literature in general [1, 2], (2) the medical literature more specifically [3–8], and (3) to a certain extent the musculoskeletal literature [7–12]. These studies have noted changes in the geographic origin of the manuscript, author gender, and an increasing number of authors. The reasons behind these changes are many and cannot be completely explained.

Efforts are being made to promote collaboration within the scientific community [13]. The scientific community has traditionally been guarded [14, 15] due to the competition between institutions for publications, funding, and scientific discovery. The advent of technology and the Internet has made it easier for researchers to collaborate with others from different institutions and countries to produce work that is mutually beneficial for all [16–20]. Utilizing the strengths of each participating institution or department can result in a product far superior than what would be achieved individually.

Publications are becoming increasingly important at all stages of academic careers from residency and postgraduate/postdoctoral applications to obtaining grants and tenure [2–36]. Manuscript publication is useful to obtain competitive residency and fellowship programs [36–38]. In the 2018 Residency Match [39], successful orthopaedic surgery applicants had an average of 11.5 unique research experiences (abstracts, presentations, and publications), compared to 6.7 for those who did not match.

Many efforts are being made to close the gender gap in all of society, including science and medicine. Women comprise approximately 50% of the world population [40, 41] but account for only 8% of top earners in professional fields [42, 43]. There has been significant discussion regarding women overcoming barriers that hinder career development. In 2014, women received a majority of doctoral degrees, yet there are far fewer women at the professor level in academia [44]. Since 1980, the fraction of women gaining doctorates in science has more than doubled in the United States and is nearing equity [45], yet representation of women among science and engineering faculty in the US lags behind the gains in graduate education. Their progression to higher faculty ranks is diminished behind that of their male counterparts, in part because many women do not apply for tenure-track jobs, even though a study of US science departments showed that women were more successful than men in gaining tenure between 2002 and 2004 [46]. Some of the gender inequalities that exist in the scientific fields include compensation and hiring differences [45], NIH grant funding rates [47], and patenting activity in the biotechnology industry [48, 49]. This is in spite of a recent study noting that University academic departments wishing to hire a new STEM (science, technology, engineering, mathematics) tenure-track faculty at the assistant professor level [50] demonstrated a 2 : 1 preference for female applicants. However, this aspiration does not currently match the reality. Within engineering, for example, in 2016, 20.9% of bachelor's degrees (BS) and 23.3% of doctoral degrees were awarded to women [51]; 16.0% of academic faculty positions were occupied by women.

Medicine has traditionally been a male-dominated field, although women have made significant gains. Women accounted for 34% of active physicians in the US in 2015 [52]; however, there is a wide range between specialties [52]. The percentage of women physicians in the US is highest in pediatrics (61.9%) and lowest in orthopaedic surgery (5.0%). In 2018–2019, women represented 49.5% of US medical school matriculates [53]. Despite this near equal representation of women graduating from medical school, the representation of women in the field of surgery is low [54], being 19.2% in general surgery, 15.0% in plastic surgery, 11.3% in vascular surgery, 8.0% in urology, 7.8% in neurosurgery, 6.0% in thoracic surgery, and 5.0% in orthopaedic surgery [55]. Using 2016-2017 data, obstetrics-gynecology, dermatology, and pediatrics had the highest percentage of women residents at 75.1%, 62.1%, and 60.4%, respectively [56], and the lowest percentages in orthopaedic surgery and nuclear medicine at 14.7% and 5.8%, respectively. Within US orthopaedic surgery subspecialty fellowships, pediatric orthopaedics [57], and hand surgery [58] had the highest percentage of women at 23% and 25% respectively, and spine the lowest at 3% [57].

In medicine, even after residency training, women have a more difficult time advancing compared to men in the same field [59–63]. Currently ∼50% of medical school graduates are women [64], yet only 21% of full-time professor positions within medicine are held by women [65]. Women comprise less than 30% of all clinical faculty, and only 15% of clinical faculty in surgical specialties [65]. Women account for only 14.7% of orthopaedic surgery residency positions in the US and 4.3% of orthopaedic surgeons at academic medical schools [56, 66].

The gender discrepancy is significant in several areas of academic medicine, including the issue of publications. The seminal study of Jagsi et al. [67] reported on authorship gender disparity in leading medical journals (*New England Journal of Medicine, Journal of American Medical Association, Annals of Internal Medicine, Annals of Surgery, Obstetrics & Gynecology,* and *Journal of Pediatrics*). Female authors increased from 5.9% in 1970 to 29.3% in 2004, and senior female authors increased from 3.7% in 1970 to 19.3% in 2004. In the 2004 Annals of Surgery, these same numbers were only 16.7% and 6.7%, respectively, confirming the fact that surgery is still a male-dominated field. Other studies [3, 68] reviewing authorship gender in their respective specialties noted underrepresentation of women as both first and principal author in ophthalmology [68] and radiology journals [3].

The musculoskeletal literature is deficient on general bibliometric and authorship gender studies, although there has been recent interest [7–11, 69–73]. With this deficiency in mind, the purpose of this study was to analyze bibliometric changes of the musculoskeletal literature (basic science, translational, and clinical) over the last 30 years. This literature is wide in scope, spanning both orthopaedic surgery and STEM disciplines. It provides an avenue to compare and contrast these two very important fields in academia within the same general topic (musculoskeletal). Bibliometric studies provide valuable information regarding past, current, and future directions in the field and are one way of understanding the impact of gender on research and how to overcome gender bias [74]. Such data are helpful for mentors in counseling trainees and junior faculty. It may also assist policy development for governing higher education and research grant awards.

## 2. Materials and Methods

### 2.1. Data Collection

During 2017 and 2018, our group performed bibliometric analyses spanning the last 30 years for select musculoskeletal journals (both basic science and clinically oriented journals, including subspecialty orthopaedic journals). Many of the results have been published for each or pairs of the journals [75–84], but not all results for all journals in a detailed manner. This study reports our comprehensive results, with comparisons between journals and journal types. The journals selected for study were the *Annals of Biomedical Engineering (ABME), Bone, Calcified Tissue International (CTI), Journal of Bone and Mineral Research (JBMR), Journal of Orthopaedic Research (JOR), American Journal of Sports Medicine (AJSM), Arthroscopy (ARTHRO), Bone and Joint Journal (BJJ) (*formerly known as the *British Journal of Bone and Joint Surgery), Foot and Ankle International (FAI), Injury, Journal of Arthroplasty (JAR), Journal of Bone and Joint Surgery (JBJS), Journal of Hand Surgery-American (JHSA), Journal of Hand Surgery-European (JHSE), Journal of Orthopaedic Trauma (JOT), Journal of Pediatric Orthopaedics (JPO),* and *Spine*. These journals are a representative sample, but clearly not exhaustive, of both the basic science and clinical orthopaedic musculoskeletal research literature. We made a conscious decision to not include physical medicine or rheumatology journals as our research group is within the department of orthopaedic surgery, with both clinical and basic science sections. The journals were grouped into two major types: primarily basic science or clinical, acknowledging that there are often overlaps between these areas for all journals. The basic science group consisted of ABME, Bone, CTI, JBMR, and JOR; the remainder comprised the clinical group.

One year from each decade was selected. For those studied in 2017, the decades were 1985, 1995, 2005, and 2015; for those studied in 2018, the decades were 1986, 1996, 2006, and 2016 (For JOT, 1987, its inaugural year, was used; and for Arthroscopy, the first two years of 1985/1986 were used in order to obtain adequate numbers). Such methodology using years separated by a decade has been previously validated [3, 4, 7. 10, 26, 30, 67, 75, 76, 85]. A PubMed search was performed for each year. Editorials, letters, and commentaries were excluded from the search, and the citations for the remaining entries were downloaded into EndNote X7 (Thomson Reuters, New York, NY, USA, 2013). The entries were viewed manually to eliminate those published electronically in the desired year but not published in hard print until the following year. All entries without authors were excluded, as well as memorandums and meeting notes. These data were extracted and placed into an Excel file in preparation for further tabulation of demographic data. The data collected were the names of the first and corresponding authors, corresponding author position (e.g. 1^st^, 2^nd^, 3^rd^,…, or last in the byline position), manuscript length (number of pages), number of references, number of times the manuscript was cited, and country of origin for the corresponding author. Citation data were obtained from a Scopus search during the month of December 2016 for the 2015 journals and December 2017 for the 2016 journals. An annualized (normalized) citation was created by dividing the citation number by the age of the manuscript in years. This corrects for the odds of a manuscript being cited the longer it has been published. For example, a manuscript published 30 years ago has a higher potential of being cited compared to one 5 years ago. This normalized citation adjusts for the odds of a manuscript being cited the longer it has been published.

Author gender was determined for the first and corresponding authors using the method described by Mimouni et al. [68]. Each author's first name was entered into “Baby Name Guesser” at http://www.gpeters.com/names/baby-names.php. This gives the most likely gender with a gender ratio. A ratio ≥3.0 is considered as a correct gender assignment. For those <3.0, a Google search was performed to determine the gender. If that was unsuccessful, the entry was excluded for gender analyses. The gender of the editors and editorial board members was also determined for each journal for each of the respective years. When the manuscript had more than one author and when the first author was not the corresponding author, a gender combination between the first and corresponding author was tabulated (MM–both authors male, FF–both authors female, MF–1^st^ author male and corresponding author female, FM–1^st^ author female and corresponding author male). This was used as an indirect method to study mentoring between/within genders.

The country of manuscript origin was grouped into regions. North America was the United States and Canada. Europe was grouped as the European continent, including Russia and Turkey. Asia was defined as all countries east of Turkey, including the Middle East and Israel. Latin America was defined as Mexico, Central America, and South America. Africa and Australia/New Zealand were the other regions. The state/province was obtained for those institutions located in the United States or Canada.

Regions within the United States were categorized as Northeast, West, South, and Midwest as determined by the US Census Bureau (https://www.census.gov/geo/reference/webatlas/regions.html). We arbitrarily categorized the regions within Canada as West (British Columbia and Alberta), Midwest (Ontario, Manitoba, Saskatchewan), and East (Quebec and those farther east). Europe was divided into regions using two different schemes. The first scheme was geographical—the British Isles (The United Kingdom, Northern Ireland), Nordic (Sweden, Denmark, Norway, Finland), and Continental (all others). The second scheme was historical—was the country a former Eastern Bloc member or similarly politically aligned (USSR, Russia, Poland, Czech Republic, Slovak Republic, Hungary, Romania, Bulgaria, Albania, and Yugoslavia with its subsequent divisions (Slovenia, Croatia, Macedonia, Serbia)). Although not technically correct, all the manuscripts from Germany were considered to be Western Bloc. Of the 379 manuscripts from Germany, 15 were in the 1985–1987 group; some of those could have originated from East Germany rather than West Germany, as the Iron Curtain fell between the years of 1985–1987 and 1995–1996. However, these small numbers would have minimal impact on the results of the Eastern Bloc status. Finally, the journals were grouped into two regions based upon the location of the editorial office: North America or Europe.

Corresponding author position was studied in three ways. The first simply describes the locations of the corresponding author as first, second, last, or other. Throughout the rest of the manuscript, this method uses “location” as the adjective. The other two use continuous variables. The first is simply the numerical position of the corresponding author in the byline of all authors. However, there has been an increase in author numbers over time. This was standardized by dividing the numerical position of the author by the total number of authors to give a normalized value. Throughout the rest of the manuscript, these two continuous variable methods use “position” as the adjective.

### 2.2. Statistical Analyses

Continuous data are reported as the mean ± 1 standard deviation. Discrete data are reported as frequencies and percentages. Analyses between groups of continuous data were performed using nonparametric tests due to the data not having normal distributions (Mann–Whitney U test—2 groups, Kruskal–Wallis test—3 or more groups). A multifactorial ANOVA was used to study the effect of various categorical variables on a continuous variable, as there is no good nonparametric test equivalent [85]. Differences between groups of discrete data were analyzed by the Fisher's exact test (2 × 2 tables) and the Pearson's *χ*^2^ test (greater than 2 × 2 tables). Trends over time for categorical variables were assessed using the Cochran linear trend test (2 × tables). Multivariate logistic regression was used to determine predictor variables of authorship gender (first, corresponding, and gender combination) and single authorship, giving odds ratios (OR), 95% confidence intervals [CI], and associated *p* values. Statistical analyses were performed with Systat 10 software™ (Chicago, IL, 2000).

The reader must be aware that when multiple statistical tests are performed on a single data set, there is an increased chance of finding a significant value when in fact it is not truly significant. In this study, ∼350 unique statistical analyses were performed. Some statisticians do not believe that a correction for multiple analyses is needed [86, 87], and in many circumstances may be counterproductive [87]. This is an area of considerable discussion in statistics [86–89]. However, many others believe that some sort of adjustment should be used. Assuming that there should be an adjustment, one method of correction is that of Holm [90]. The Holm correction for these 350 analyses gives a *p* < 0.0005 of being statistically significant. This is extremely conservative, and we suggest that for our type of study such a limit will exclude important information. This is not a clinical trial looking at outcome measures [87]. Another approach is to simply set the *p* value lower, such as 0.005; this has been recently proposed by major journals [91, 92]. These authors, however, note that this is meant for very important decisions regarding new medical treatments. Clearly when a new medical treatment is being considered, it should be strongly proven. However, in this study, we are not making any inferences regarding new treatments but rather performing exploratory studies in bibliometrics and how it relates to author gender between various variables. Thus, it is possible that the 0.005 is too conservative; however, we have elected to use this value and consider those between 0.005 and 0.05 as suggestive [92]. Throughout this study, the actual (not Holm adjusted) *p* value is given, allowing the individual reader to decide appropriate significance.

## 3. Results

### 3.1. Overall Results

There were 12,819 manuscripts that met the inclusion criteria; 3,178 (24.8%) were in the basic science and 9,641(75.2%) in the clinical groups. The journal was based in North America for 9,857 (76.9%) and in Europe for 2,962 (23.1%) manuscripts. With regard to published manuscripts, the numbers were 1,576 (12.3%) in 1985–1987, 2,830 in 1995–1996 (22.1%), 3,924 in 2005–2006 (30.6%), and 4,489 in 2015–2016 (35.0%). The gender of the 1^st^ author was male in 10,191 (79.5%), female in 2,210 (17.2%), and unknown in 418 (3.3%) manuscripts. The corresponding author was identified in 12,692 manuscripts; the gender of the corresponding author was male in 10,555 (83.2%), female in 1,782 (14.0%), and unknown in 355 (2.8%). Author gender combination was known for 4,557 manuscripts. It was MM in 3,174 (69.7%), MF in 367 (8.1%), FM in 777 (17.1%), and FF in 239 (5.2%). The region of origin was known for 12,816 manuscripts: North America (6,651–51.9%), Europe (3,846–30.0%), Asia (1,771–13.8%), Australia/New Zealand (393–3.1%), Latin America (93–0.7%), and Africa (62–0.5%). Due to the small numbers from Africa and Latin America, they were excluded from detailed regional analyses.

The average number of authors was 4.7 ± 2.4, institutions 2.0 ± 1.5, countries 1.2 ± 0.6, references 28.6 ± 19.2, pages 6.7 ± 2.9, citations 32.0 ± 55.8, annualized citations 2.98 ± 4.04, corresponding author position 2.3 ± 2.3, and normalized corresponding position 0.51 ± 0.34 (Table 1). There were differences between the 17 journals for all the above variables. The basic science group demonstrated greater values for all these variables compared to the clinical group. North-American-based journals had equivalent or larger values for all these variables compared to European journals except for the normalized corresponding author position. All variables increased over time except for the number of annualized citations, which dropped in the 2015–2016 decade. There were differences in all variables by region, with Asia having the highest number of authors and corresponding author positions; Australia/New Zealand the highest number of institutions and references; North America the highest number of pages, normalized corresponding author positions, and citations; and Europe the highest number of countries. Those with a female first author had more authors, institutions, countries, references, pages, annualized citations, and corresponding author position but with a lower normalized corresponding author position. Those with a female corresponding author had more authors, institutions, countries, references, pages, annualized citations, a lower normalized corresponding author position, and no difference in the unadjusted corresponding author position.

The decade, journal/journal type, author, gender, and geographic region were the variables entered into the multifactorial ANOVA (Table 2) to study their effects on continuous bibliometric variables. When using each specific journal, author number was dependent on all variables except the corresponding author gender. The number of institutions, countries, and references were dependent upon the decade, journal, and geographic region of origin. The number of references was dependent upon the decade, journal, and geographic region. The number of pages was dependent upon the decade, journal, 1^st^ author gender, and geographic region. The number of citations and annualized citations were dependent upon the decade, journal, and geographic region. The corresponding author position, both actual and normalized, was dependent upon all variables.

When using journal type (basic/clinical) instead of the actual journal, the author number was dependent upon all variables except for the corresponding author gender. The number of institutions, countries, pages, and references were dependent upon decade, journal type, and geographic region of origin. The number of citations and annualized citations were dependent upon the decade and geographic region. Both the actual and normalized corresponding author positions were again dependent upon all the variables.

### 3.2. Gender Analyses

There were differences in first author gender by journal type, specific journal, decade, geographic region, corresponding author location, and single authorship (Table 3). There were more female 1^st^ authors in the basic science group compared to the clinical group (33.2% vs. 12.7%). The percentage of female first authors rose from 10.8% in 1985–1987 to 23.7% in 2015–2016. The highest percentage of female first authors was in Australia/New Zealand (29.9%) and the lowest in Asia (13.6%). The corresponding author was more commonly last compared to 1^st^ (23.6% vs. 15.5%) when the first author was female. Single-authored manuscripts were more likely to be written by males (5.1% vs. 2.4%). There were differences in corresponding author gender by journal type, specific journal, decade, and geographic region. Female corresponding authors were more common in the basic science group compared to the clinical group (25.8% vs. 10.6%). The percentage of female corresponding authors rose from 8.9% in 1985–1987 to 18.9% in 2015–2016. The highest percentage of female corresponding authors was in Australia/New Zealand (23.3%) and the lowest in Asia (10.8%). There were no differences between North-American- and European-based journals by author gender, either 1^st^ or corresponding (*p* = 0.33 and *p* = 0.36, respectively).

**Figure 1:**
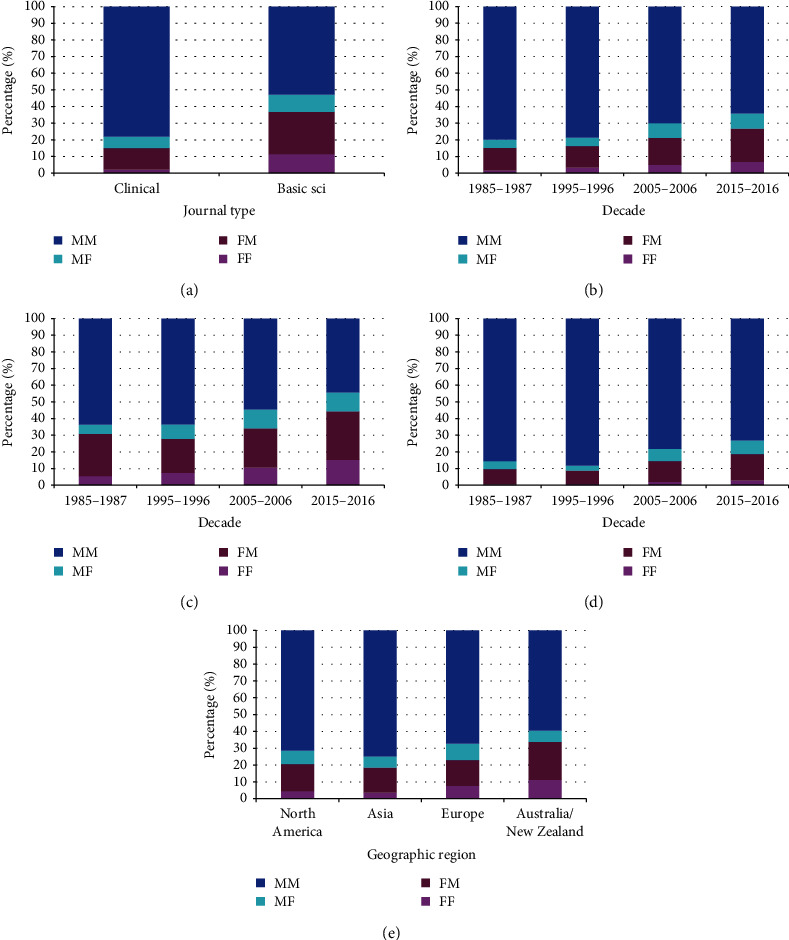
Gender combinations of first and corresponding authors, excluding single-authored manuscripts and those where the first author was also the corresponding author (FF = both authors female, FM = 1^st^ author female and corresponding author male, MF = 1^st^author male and corresponding author male, MM = both authors male). (a) By journal type (*p* < 10^−15^). (b) By decade (*p* = 1.4 × 10^−14^). (c) By decade for basic science journals (*p* = 1 × 10^−6^). (d) By decade for clinical journals (*p* = 2.5 × 10^−10^). (e) By geographic region (*p* = 1.1 × 10^−7^).

For author gender combinations, there were more FF and FM (Table 4) in the basic science vs. clinical science journals (Figure 1(a)), more recent decades (Figure 1(b)), and Australia/New Zealand compared to other regions (Figure 1(c)). There were no differences between North-American- and European-based journals by author gender combinations (*p* = 0.57).

### 3.3. Changes over Time

**Figure 2:**
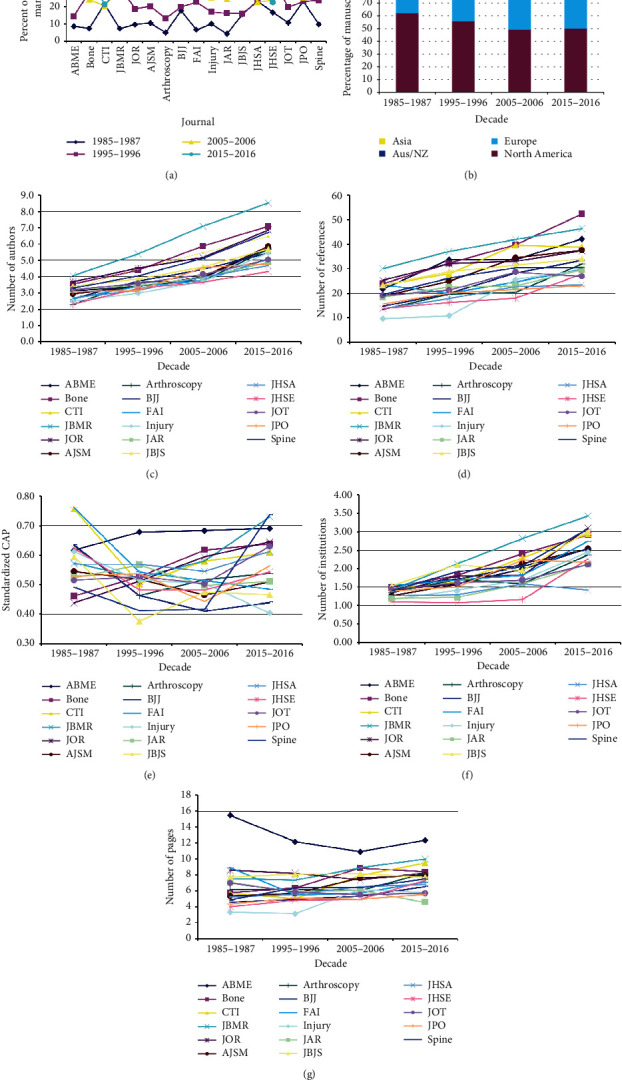
(a) The percentage of manuscripts published per year by specific journal (*p* < 10^−15^). ABME = *Annals of Biomedical Engineering*, CTI = *Calcified Tissue International*, JBMR = *Journal of Bone and Mineral Research*, JOR = *Journal of Orthopaedic Research*, AJSM = *American Journal of Sports Medicine*, BJJ = *Bone and Joint Journal*, FAI = *Foot and Ankle International*, JAR = *Journal of Arthroplasty*, JBJS = *Journal of Bone and Joint Surgery*, JHSA = *Journal of Hand Surgery American*, JHSE = *Journal of Hand Surgery European*, JOT = *Journal of Orthopaedic Trauma*, and JPO = *Journal of Pediatric Orthopaedics*. (b) Changes over time by region of origin for all manuscripts (*p* < 10^−15^). (c) Increasing number of authors over time for all journals (*p* < 10^−15^). (d) Increasing number of references over time for all journals (*p* < 10^−15^). (e) Change in the standardized corresponding author position over time for all journals (*p* < 10^−15^). (f) Change in the number of institutions over time for all journals (*p* < 10^−15^). (g) Changes in the average number of pages per manuscript over time and by journal (*p* < 10^−15^).

Of the 17 journals, seven demonstrated an increased percentage in the number of manuscripts per decade (BONE, JOR, AJSM, Injury, JAR, JHSA, JOT), five had essentially no change between 2015 and 2016 compared to 2005-2006 (ABME, CTI, JBMR, JHSE, JPO), and five (Arthroscopy, BJJ, FAI, JBJS, Spine) had fewer manuscripts in 2015–2016 compared to 2005–2006 (Figure 2(a)) (Table 5). Proportionally, manuscripts from North America and Europe decreased over time (Figure 2(b)) while manuscripts from Asia demonstrated an increase. There was an increase in author numbers for all 17 journals, going from 3.0 ± 1.5 in 1985–1987 to 5.8 ± 2.7 in 2015–2016 (Figure 2(c)). The percentage increase was the highest for Arthroscopy (147%) and the lowest for JAR (53%). There was an increase in the number of references per manuscript over time for all journals (Figure 2(d)), going from an average 18.3 ± 13.2 in 1985–1987 to 33.9 ± 20.4 in 2015–2016. Some journals had greater increases compared to others. Injury had the highest increase (204%) and FAI the lowest (27%). The standardized corresponding author position increased over time in some journals, indicating a move toward the end of the author byline, while in other journals it did not change or moved in the opposite direction over time (Figure 2(e)). The journal with the greatest move to the end of the author byline was *Spine* (50%), going from 0.49 to 0.74, the greatest move in the opposite direction was in *FAI* (36%), going from 0.76 to 0.48. Overall, eight of the journals moved more to the end of the byline while nine moved more to the beginning of the byline. The number of institutions involved in a study increased over time, going from 1.4 ± 0.7 in 1985–1987 to 2.6 ± 1.9 in 2015–2016, and was seen in all journals (Figure 2(f)). The number of pages on average increased over time, going from 5.8 ± 3.3 in 1985–1987 to 7.3 ± 2.8 in 2015–16; however, there was marked variability by journal (Figure 2(g)) with 11 showing an increase, five a decrease, and one (JBJS) with no change. The journal with the greatest increase in page number was JHSE (82.1%) and the journal with the greatest decrease was JAR (−35%). The raw data for Figures 2(b)–2(g) are given in S1 Appendix.

### 3.4. Corresponding Author Location

Corresponding author location was known for 12,382 manuscripts. It was first in 60.4% (7,479), second in 8.2% (1,020), other in 3.4% (421), and last in 28.0% (3,462). We deleted the second and other positions for further analyses (Table 6), keeping the first and last positions. The corresponding author occupying the last position differed markedly by journal, ranging from 18.5% to 52.1%, and was 46.1% for the basic science and 26.7% for the clinical journals. The corresponding author occupying the last position increased over time, going from 16.9% in 1985–87 to 44.1% in 2015–2016 (*p* < 10^−15^). The corresponding author occupying the last position was observed in 20.8% of the manuscripts from Europe and 37.6% from North America (*p* < 10^−15^). Of note, the first author was more commonly the corresponding author in European-based journals compared to North-American-based journals (78.2% vs. 65.3%, *p* < 10^−15^).

### 3.5. Single Author Manuscripts

The percentage of single author manuscripts was 4.8% (621 of 12,819) and ranged from 0.9% in JBMR to 9.6% in JHSE (Table 7). Single-author manuscripts declined over the past 30 years from 14.0% to 1.6% (*p* < 10^−15^). Single authorship was more common for manuscripts originating from Europe (5.4%) compared to other regions (3.0 to 4.9%) (*p* = 0.00017), was more common in the clinical journals compared to the basic science journals (5.5 vs. 2.8%, *p* = 6.1 × 10^−10^), and was more common in European-based journals compared to North-American-based journals (6.8 vs. 4.3%, *p* = 1.1 × 10^−8^).

### 3.6. Analyses within Regions

This section focuses on findings within regions as the more global results are described above. Of the 6,651 manuscripts from North America, 6,120 (92.0%) came from the United Sates and 531 came (8.0%) from Canada; the four most common states were California (761–12.4%), New York (642–10.5%), Pennsylvania (418–6.8%), and Massachusetts (407–6.7%). There were 1,737 from the North East (28.4%), 1,644 from the Midwest (26.9%), 1,534 from the South (25.1%), and 1,199 from the West (19.6%) (S2 Appendix). There were some significant differences between the different regions (decade, journal type, first author gender), but when visually reviewing them they are likely not meaningful.

For Canada, the four most common provinces were Ontario (285–60.0%), Quebec (90–17.7%), Alberta (56–11.0%), and British Columbia (55–10.8%); 69.5% were from the Midwest, 19.6% the East, and 10.8% the West. It was suggestive that the East was more likely to contribute a basic science manuscript (44.0% basic science, 56.0% clinical) compared to the West (30.9% basic science, 69.1% clinical) and the Midwest (27.4% basic science, 72.6% clinical) (*p* = 0.0066) (S3 Appendix). The East also had the lowest percentage of MM gender author combination (42.2%) compared to the West (53.6%) and the Midwest (66.5%) (*p* = 0.005).

**Figure 3:**
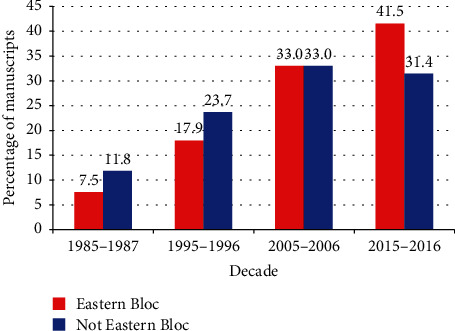
Differences by decade for previous Eastern Bloc nations (*p* = 0.013—Cochrane linear trend test). The sum of the percentages for each group will equal 100. Thus, the percentage of manuscripts coming from Eastern Bloc countries in 2015–2016 accounted for 41.5% of all the manuscripts from the Eastern Bloc.

Of the 3,846 from Europe, the four most common countries were the United Kingdom (1,360–35.4%), Germany (379–9.9%), The Netherlands (269–7.0%), and France (254–6.6%). The manuscripts from the Nordic countries and British Isles (S4 Appendix) decreased over time and those from Continental Europe increased over time (Continental Europe went from 6.2% in 1985–1987 to 28.3% in 2015–2016 (*p* < 10^−15^)); the percentage of basic science manuscripts ranged from 14.7% for the British Isles to 32.7% for Nordic Europe (*p* < 10^−15^); single-author manuscripts ranged from 3.0% for Continental Europe to 8.0% for the British Isles (*p* < 10^−9^); female first author manuscripts ranged from 16.7% for Continental Europe to 25.3% for Nordic Europe (*p* = 0.00004); female corresponding author manuscripts ranged from 13.1% for Continental Europe to 23.5% for Nordic Europe (*p* = 1.2 × 10^−7^); and MM gender author combination ranged from 57.9% in Continental Europe to 70.1% in the British Isles (*p* = 0.0008). When analyzing by the political division groups in Europe, 2.8% came from the Eastern Bloc. The only noticeable difference between the Eastern Bloc and the remainder of Europe (S4 Appendix) was the number of Eastern Bloc manuscripts over time (Figure 3). Of all the manuscripts from the Eastern Bloc, 7.5% were published in 1985–1987 and increased to 41.5% in 2015–2016; or stated differently, 1.8% of all manuscripts in 1985–1987 came from the Eastern Bloc and doubled to 3.6% in 2015–2016.

Of the 1,771 published manuscripts from Asia (S5 Appendix), 1,463 originated from four Asian countries: 39.6% (702) originated from Japan, 18.9% (334) from China, 15.6% (276) from South Korea, and 7.0% (124) from Israel. There were marked differences between all four countries (all *p* < 10^−6^) except for single-author manuscripts (*p* = 0.75). The proportion of manuscripts from China and Korea increased over time while the proportion from Japan decreased and from Israel it was relatively unchanged. Of all the manuscripts from China, 0.9% were published in 1985–1987 and increased to 75.4% in 2015–2016. A similar trend was noted for those from Korea, going from 0% of their total in 1985–1987 to 64.1% in 2015–2016. For Japan, the percentage of all their manuscripts went from 7.5% in 1985–1987 to 31.6% in 2015–2016; the percentages for Israel remained relatively constant, the low being 23.4% in 1985–1987 and the high being 27.4% in 2005–2006. The percentage of manuscripts in basic science journals per country was highest for Israel (33.1%) and lowest for Korea (13.0%). The percentage of female first and corresponding authors was highest for manuscripts from China (21.5% for both) and lowest for Japan (8.3% and 6.1%, respectively). The author gender combination MM was lowest in China (58.1%) and highest in Japan (86.7%).

### 3.7. Predictors of a Female Author and Single Author Manuscript

We next determined which variables could predict author gender or a single author manuscript. For female-authored manuscripts, two different analyses were performed. One included each journal individually, and the other used journal type (Table 8). The odds ratio (OR) of a female first author was highest for CTI (7.9 [5.6, 11.2]) and lowest for Arthroscopy (1.0), the present decade compared to prior decades (OR 2.9 [2.4, 3.5]), and Australia/New Zealand (OR 2.7 [2.0, 3.6]). The OR of a female corresponding author was highest for JBMR (OR 6.6 [4.6, 9.5]), present decade (OR 2.5 [2.1, 3.1]), and Australia/New Zealand (OR 2.4 [1.7, 3.2]). When analyzing by journal group, the findings for decade and region were exactly the same but with slightly different ORs. By journal type, the OR of a female first author was higher in the basic science group compared to the clinical group (OR 3.5 [3.2, 3.9]) and was the same for a female corresponding author (OR 3.0 [1.6, 2.3]).

For single-authored manuscripts, two different analyses were performed. The first excluded author gender and the second included author gender (Table 9). In general, the results were very similar for both analyses. Single-authored manuscripts were most common in Injury and Arthroscopy and least common in JBMR, had a male author, were from the 1985–1987 decade, and were from a clinical journal.

### 3.8. Editorial Board Changes by Gender

**Figure 4:**
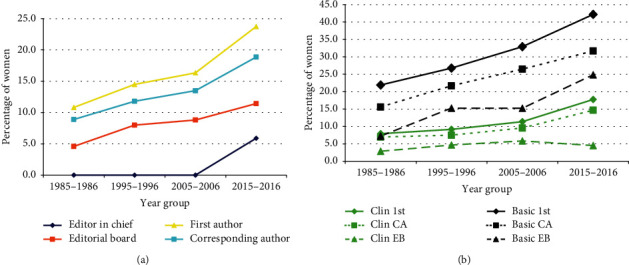
Changes over time in the percentage of women in the musculoskeletal literature by role. (a) The percentage of women being a first author, corresponding author, journal editorial board member, or editor in chief. (b) Changes by the type of musculoskeletal journal (Clin = clinical, Basic = basic science, 1^st^ = first author, CA = corresponding author, EB = editorial board).

While analyzing changes in author gender, we also wished to determine if such changes reflected the editorial board composition and compare that to the first and corresponding authors (Table 10). The author gender paralleled that of the editorial board composition. That is, as the number of female first authors increased, so did the number of female corresponding authors, editorial board members, and editors in chief. Over time, the greatest number of women was in the first author position, followed by the corresponding author, editorial board, and editor in chief position (Figure 4(a)). The clinical group of journals consistently lagged behind the basic science group (Figure 4(b)).

## 4. Discussion

As with any study, there are limitations. First, the accuracy of our gender-based analysis depends on the accuracy of the website for gender ratio scores ≥3.0. However, this website/technique has been previously validated [68]. We also analyzed one year per decade. While we recognize this is not an analysis of all data for every year, the decade method was compared to a 10% random sampling of all manuscripts from each year in JBMR [75], and there were no significant differences based on the method used. As a result, we are confident that the decade approach is reliable for these types of bibliometric studies. Another limitation is that all of the journal's editorial offices are based either in North America or Europe, which lends our study's bias to those regions. We excluded much from the global south because the lower numbers were not sufficient to make meaningful analyses, and this lack of representation of authors within these journals could mean that there is a gap in worldwide knowledge of musculoskeletal publishing trends from those areas of the world.

This is obviously not a comprehensive study of the entire English musculoskeletal literature. There are many more musculoskeletal journals that could have been studied as well. However, time and human resource constraints limit the number of such journals, as the acquisition of these data are extremely time-consuming and much is manually curated. We apologize if a certain journal was not included in these analyses, as we were trying to study a wide sample of the musculoskeletal literature, both basic science and clinical, from both the North American and European continents, and both general and subspecialty orthopaedic surgery journals. Author ethnicity was not studied, as we could not identify any appropriate, validated means to obtain such data; we acknowledge this would have been an interesting aspect to study.

With these limitations in mind, we noted many interesting findings. First, one-half of the journals had an increase in the number of manuscripts published per year, while in the other one half it dropped. One explanation may be the increasing number of journals in which an author may publish [93, 94], especially subspecialty orthopaedic journals. Some of the journals have recently developed sister journals within their own journal as well (i.e., for JBJS, besides the flagship JBJS, they are also publishing JBJS Essential Surgical Techniques, JBJS Reviews, JBJS Case Connector, and JBJS Open Access). Next is the increasing number of authors per manuscript. This has been noted by others [9, 21–24, 27–29]. It was projected that by 2034 the average paper will list 8 authors [28]. The importance of publications in career advancement in academia likely explains this finding. Some individuals accept unearned authorship [95, 96], and even in the most influential medical journals, prevalence of ghost/honorary authorship is estimated to be 21%. Using 2–3 authors per article as baseline, 4–6 coauthors increase the chance of honorary authors by 3.5; 7–10 by 7.9; and 11 or more by 10.8. Power asymmetry among coauthors leads to this phenomenon, as powerful senior researchers often simply read their junior colleague's manuscript, and by approving it, feel entitled to authorship [97]. Indeed, Kovacs states that, “without ensuring a really democratic framework for authorship decisions, the law of the jungle prevails, as often is the case today in publication science.” Finally, a 2020 study of the surgical literature regarding courtesy authorship and different generations of surgeons [98] noted that both junior and senior faculty publishing in the surgical literature had similar historical rates of adding a courtesy author (58% junior, 51% senior) and that junior faculty more frequently added a courtesy author compared to senior faculty (23% versus 13%). The junior faculty felt more pressure by superiors to add courtesy authors, although interestingly senior faculty stated the reason to add courtesy authors was to avoid conflicts more frequently than junior faculty (33% vs. 17%). As long as such behavior prevails, there will likely continue to be an increasing number of authors per manuscript.

The musculoskeletal literature does not demonstrate the massive numbers of authors that occurs in other areas of science. Mega-authored manuscripts are common in the very complex world of high-energy physics [99]. The most authors in the world on a manuscript, to the best of our knowledge, is 3,173, and involved a study from the Large Hadron Collider project; indeed, most manuscripts with over 1000 authors are from that same project [28]. There are differing opinions regarding author inflation. Some believe that if it is due to increasing study complexity, then nothing is inherently objectionable to increasing authorship [28]. The rising number of authors can also be interpreted as a positive phenomenon, reflecting increasing collaboration [23, 95, 100–105]. However, that view is not held by all orthopaedic surgery journals. Indeed, in the past few years, several orthopaedic journals have limited the number of authors allowed on a manuscript (JBJS, JAR, and JHS no more than 6, Arthroscopy no more than 7, and for AJSM only 12 will be placed on the masthead). The impact of such restrictions will become evident several years from now and will likely decrease the number of authors for those particular journals. However, it may result in an even lower number of submissions and subsequent publications for such a journal as some researchers may submit their study to a different journal having no limitation on the number of authors, believing that with the increasing complexity of research, more participants/authors are needed to perform the research. An author number restriction may not appropriately credit all authors as defined by the International Committee of Medical Journal Editors [106].

The increasing number of authors per manuscript seen in this study likely represents increasing collaboration, supported by the increasing number of institutions and countries over time. Advancements in technology have allowed much easier collaboration between institutions and countries. Researchers can now access manuscripts from other institutions and countries, which was more difficult before the ubiquitous nature of the Internet. The increasing number of references per manuscript is likely attributed to this ease of identifying other relevant publications due to advances in computer search capabilities and access to multiple databases. There are many advantages to collaboration including resource sharing, allowing individuals with different skills to come together to solve a problem, and increasing research productivity [19, 20, 107]. Collaboration has been described as the fourth age of research [13]. There are, however, drawbacks to collaboration from a global perspective, especially when both developing and developed countries are intertwined [19, 108]. These drawbacks are: equal opportunities for all researchers, competence of potential partners, respect between all researchers involved, trust and confidence, and justice and fairness in collaboration. The regional differences seen for author number, number of institutions, countries, and references may reflect different cultural views on collaboration between regions. It would appear that female investigators are more collaborative in the musculoskeletal arena than male investigators, since the number of institutions and countries was greater for female first authors than male first authors.

While the number of authors increased, the number of single-author manuscripts concomitantly decreased from 14.0% in 1985–1987 to 1.6% in 2015–2016. This likely is due to increasing collaboration and complexity of the studies. In the clinical group, the percentage of single-author manuscripts was 5.5% and 2.8% in the basic science group. It is clearly easier for one researcher to perform a clinical case series study compared to a laboratory, basic science study.

We also noted an increase in the number of references per manuscript over time. The explosion of scientific literature [93, 94] as well as the increasing ability to access such literature via the Internet and electronic libraries likely is one factor behind this increase. Recently, some journals have been asking authors to limit the number of references. This is likely due to the need to minimize the number of pages and reduce weight/shipping costs for standard print journals. For journals that are solely digital, such a requirement would not be necessary. The same is true for the number of pages over time, with some journals showing an increase and others a decrease. As of November 2020, there were word limits for many journals (JBJS—3000, BJJ—4000, JPO—2500, Spine—2700, JOT—3000, FAI—4000, JHSA—3000, Arthroscopy—4000, AJSM—6000, JOR—420, CTI—5000), with most excluding references in these limits except for BJJ and AJSM, which included references in the word limit. Reference limits have been placed by AJSM (60), JOR (50), CTI (45), and JHSE (limit to pertinent ones).

Many changes/differences in the corresponding author position/location were noted. The normalized corresponding author position (which accounts for the total number of authors in a manuscript) was higher in the basic compared to the clinical journals (0.58 ± 0.38 vs. 0.48 ± 0.33). It was highest for those from North America (0.55 ± 0.35) and lowest for those from Latin America (0.38 ± 0.28). In some journals, it increased over time, indicating a move toward the end of the author byline, while in other journals it did not change over time (Figure 2(e)). The journal with the greatest move to the end of the author byline was JOR (66%), going from 0.39 to 0.65; the greatest move in the opposite direction was in FAI (−36%), going from 0.75 to 0.48. Overall, eight of the journals moved more to the end of the byline, while nine moved more to the beginning of the byline.

Corresponding author location was last in 26.7% of the clinical journals and 73.3% of the basic science journals. From 1985 to 87 to 2015–16, it moved from 16.9% to 44.1% (*p* < 10^−6^, CLT). For the basic science journals, the change was from 22.1% to 58.7% (*p* < 10^−6^, CLT) and for the clinical journals from 15.5% to 39.3% (*p* < 10^−6^, CLT). First authors traditionally have performed much of the research and manuscript preparation [109–114], while corresponding authors are typically the more senior person who generated the research idea or in whose clinical division/laboratory the research was undertaken. This is especially so for those studies that were more basic science in nature and is confirmed by our results. In clinical journals, even today, the first author is most commonly the corresponding author. It would appear from these analyses that the investigators in the basic science compared to the clinical realms differ in assignment of first and corresponding author locations. It is very possible that in the clinical studies, the first author was the clinician who conceived the study, did most of the surgical procedures, and drafted the manuscript, while the other authors played additional roles, such as reviewing charts/collecting data/analyzing data, and finalizing the manuscript.

There were many changes in the geographic regions of origin. Within Europe, the percentage of contributions decreased from the British Isles and Nordic countries and increased for the remainder of Europe. This is likely due to the rapid increase in publications from former European Eastern Bloc nations (Figure 3). Within Asia, there was a significant change with increases in numbers from South Korea and China (S5 Appendix). China is known to be a rapidly rising research nation [115]. This likely is due to many factors. The advent of the Internet and other easy means of communication allows for increased collaboration. Secondly, many of these countries having an increasing research/publication presence have undergone sociopolitical changes. The Eastern Bloc is now dissolved, and China and South Korea have become more important in the political spheres of the world. Within the United States, there were minimal changes in the proportion of manuscripts from the four major regions (S2 Appendix). However, every decade saw an increase in the number of papers from every region. Within Canada, there were an increasing number from Western Canada (likely British Columbia) (S3 Appendix).

Between the two major journal groups, the North East US produced more of the basic science manuscripts compared to the other three regions (S2 Appendix); this may in part be due to this region having three out of four of the most prolific states in this study. For Canada, the Midwest region (S3 Appendix) produced more of the basic science manuscripts compared to the other two regions. Within Europe, the Nordic countries had proportionally more basic science manuscripts than clinical manuscripts, with the opposite being true for the British Isles (S4 Appendix). Within Asia, Japan had the highest number of both basic and clinical science manuscripts, with South Korea contributing the lowest proportion of basic science manuscripts and Israel the lowest proportion of clinical manuscripts (S5 Appendix).

Many differences were noted for author gender, in both the basic science and clinical groups. First, the basic science journals had a greater proportion of women first and corresponding authors (33.2% vs. 12.7%-first; 25.8% vs. 10.6%-clinical) (Table 3). This is likely due to the fact that the clinical group comprises primarily orthopaedic surgery journals, and it is well-known that orthopaedic surgery is still a male-dominated field [116–124]. Many of the basic science journals involve the study of bone and mineral metabolism and bioengineering, and it is known that these areas have many more women, with primarily PhDs and not MDs. The proportion of women gaining doctorates in science has more than doubled in the United States since 1980 and is now nearing equity [45]. In biomedical engineering, in 2015, women received 40.9% of the B.S. degrees as compared to 13.2% and 12.5% in mechanical and electrical engineering, respectively [125]. Women in biomedical engineering have also received more M.S. and Ph.D. degrees than in traditional engineering areas [125].

Both journal groups demonstrated an increasing number of women authors (Table 3). The percentage of women first authors increased from 10.8% in 1985–1987 to 23.7% in 2015–2016. For the basic science group, it increased from 21.9% to 42.0% (*p* < 10^−6^), and for the clinical group from 7.9% to 17.8% (*p* < 10^−6^). There were differences by geographic region. The proportion of women first authors was highest for those from Australia/New Zealand (29.9%) and lowest from Asia (13.6%); the same pattern was true for corresponding authors, with Australia/New Zealand the highest (23.3%) and Asia the lowest (10.8%). Within Asia, there were differences, with China contributing the greatest number of women as first and corresponding authors (S5 Appendix). This is encouraging regarding the issue of gender parity in the musculoskeletal literature. The same phenomenon has been noted in the surgical literature [126–128] over the last two decades, as well as in the neurosurgery literature [129] and thoracic surgery literature [130], but not in the oral/maxillofacial [131] or hepatopancreaticobiliary [132] literature. This is different from what has been previously observed in the general medical literature. For example, Filardo et al. [64] found that representation of women among first authors in high-impact medical journals increased significantly over the past 20 years but plateaued and even declined in some journals in recent years.

One metric of studying mentorship for authors is to reflect upon the identity of the first and corresponding author. We assessed changes over time for those manuscripts having more than one author and where the first author was also not the corresponding author. Previous studies have shown that women authors prefer to be mentored by women [133]. In orthopaedic surgery, there are fewer women in the senior academic positions compared to the basic sciences. This likely explains the fact that the FF gender combination is ∼5.5 times less in clinical journals compared to basic science journals (2.1% vs. 11.5%). Only 6.8% of the clinical and 10.5% of the basic science manuscripts had a male first author and female corresponding author, likely indicating that males rarely choose female mentors (understanding that the supply of female mentors is low). Hopefully, such mentoring will continue, especially between genders. However, there is some anxiety among potential male mentors in taking on female mentees in the #MeToo era [134–136]. This has been noted by the American Academy of Orthopaedic Surgeons, resulting in education from male orthopaedic surgery leaders on how to mentor in the #MeToo era [137].

As the number of female orthopaedic surgeons is the lowest of all the surgical specialties in the United States, avenues to increase this number have been proposed. Strong mentorship was the largest modifiable factor in choosing a subspecialty for female orthopaedic surgeons [58]. Mentoring may also further help in diversifying the racial/ethnic mix of the orthopaedic surgeon workforce. The efforts to increase diversity in the orthopaedic workforce should begin early in medical school [138, 139]; an emphasis should be placed on female medical students having a positive experience on orthopaedic surgery rotations [140]. In one study, the few female applicants to orthopaedic surgery residencies strongly considered the presence of other female residents and faculty, program reputation for gender diversity, and their personal interactions with members of the programs as important factors when selecting their final residency program [141]. In another study, increased exposure to orthopaedic content and increased female mentorship might help recruit more females into the orthopaedic surgery workforce [142]. However, there are some data that these efforts have had little effect on increasing the number of female orthopaedic residents [143]. Another study noted that the number of female orthopaedic faculty did not influence the number of female applicants for orthopaedic residency [144]. Finally, such endeavors might need to occur before medical school [145], as 51% of medical students selecting orthopaedics had decided on this before their third-year rotations, with 27% deciding before medical school. However, requiring instruction in musculoskeletal medicine in medical school curriculums results in a 12% higher rate of applications to orthopaedic surgery residency [146], and was even more pronounced among women with an 82% higher rate of application to orthopaedic surgery residency.

Unfortunately, a recent study by Holman et al. [147] noted that the gender gap appears likely to persist for generations, particularly in surgery, computer science, physics, and math, unless efforts are made to effect change. They calculated the time in years to gender parity for all authors in studies for many different disciplines. They noted that gender parity will take over 100 years for physics and astrophysics, 73 years for biomedical engineering, 60 years for mathematics, 42 years for chemistry, 27 years for biochemistry, and 13 years for cell biology. Within the medical disciplines, they noted that pediatrics and obstetrics/gynecology are at gender parity, 98 years for orthopaedics, 86 years for urology, 56 years for emergency medicine, 52 years for general surgery, 42 years for traumatology, 40 years for neurosurgery, 39 years for ophthalmology, 29 years for otoloaryngology, and 8 years for rheumatology. Interestingly, physical medicine and rehabilitation was the only discipline that has a negative parity (more women than men), and they calculated that gender parity will take 34 years.

It has been noted that female scientists have a larger number of collaborators than male scientists [148, 149]. We noted that the number of authors, institutions, and countries was slightly higher for female authors compared to male authors, both first and corresponding (Table 1). This likely supports the study of Bozeman [148]. We also noted that the number of references and pages was higher for female authors (Table 1). While our study does not address the reasons for these differences, it is possible that female authors place an increased number of authors on their manuscripts in an effort to increase collaboration or are more generous in giving credit to those involved in the study. Female authors may also not be as confident that their work will be accepted due to the male predominance in orthopaedic surgery, and therefore may feel the need to place more mentors, contributing authors, and references on their work for corroboration and to support their research.

Author gender changes over time are clearly reflected in the composition of the editorial board makeup. It is standard knowledge in academia that individuals begin their academic career by becoming first authors, and then corresponding authors (as discussed above). Editorial members of a journal, after demonstrating academic productivity, as a first and subsequently corresponding author, may then be appointed to an editorial board on a journal of their discipline. From the editorial board members, the editor-in-chief is often later selected. This study demonstrated a very parallel track for these four different levels in academia. Over time, the greatest number of women was in the first author position, followed by the corresponding author, editorial board, and editor-in-chief position (Figure 4(a)). This is very encouraging and seems to demonstrate that the gender gap is being slowly closed. A recent study [150] noted that women were represented equally or in greater numbers as editors in JBJS, *Journal of the American Academy of Orthopaedic Surgeons*, and Clinical Orthopaedics and Related Research. They noted the same lag that we did when comparing editors to authors, with the percentage of female editors in 2017 roughly equaling the percentage of female authors in 2007. Regarding professional orthopaedic subspecialty societies, there was a strong correlation between the percentage of women in a society and the percentage of women on the society's board of directors [151].

Author diversity is important in that manuscripts with diverse authorship have increased citations [13]. Women who obtain a patent, especially those in academia, have a higher number of International Patent Classification codes than men. Medical manuscripts having female authors have an increased probability of reporting sex in their results [152], and there has been a recent outcry from the orthopaedic surgery community that research needs to give more gender-specific outcomes [4, 153]. In the general surgical literature, female authors proportionally include more female participants in their studies, and studies addressing sex differences have increased citation rates [154]. Thus, this is another benefit of having more female authors, as it will likely improve the quality of care for female patients.

The regional differences seen in many of the bibliometric variables (author number, number of institutions, countries, references, author gender) likely represent different cultural views between regions. It is well-known that different parts of the world have markedly different percentages of male and female physicians. The percentage of female physicians is the highest in Latvia (74.3%) and the lowest in Japan (20.3%) [155], although the figures were not broken down by medical specialty. Some societies are more patriarchal, while others are more matriarchal [156]. This could possibly influence the differences in author gender noted in this study. While Saudi Arabia has been accused of treating women poorly, according to the Human Rights Watch organization [157], the *Journal of Musculoskeletal Surgery and Research* (the official journal of the Saudi Orthopaedic Association) was noted to have more female authors compared to the *Egyptian Orthopaedic Journal* [158]. The prevalence of international contributions was higher in the JMSR compared with the *Egyptian Orthopaedic Journal*. Thus, it is difficult to state that perceived gender differences/rights/abilities are seen in the musculoskeletal research productivity area.

Regarding future research, one important area is to study changes in the race and ethnicity of authors over time. Such data are difficult to acquire unless each and every author can be contacted and they themselves self-identify their race/ethnicity. Perhaps, this could be done for corresponding authors, as that contact information is given in most manuscripts, but it would be difficult to know the response rate for such questioning. Another area of further research would be to explore changes in journals published in languages other than English, such as Spanish in journals from Mexico, Central and South America; Chinese in journals from China; Japanese in journals from Japan, etc. In addition, since all the journals highlighted in our research have editorial boards in either North America or Europe, expanding to journals with editorial boards in the Global South or Asia may also provide a broader perspective. Such studies might give further insight into some of the differences between geographic regions that we noted in this study.

## 5. Conclusion

In this detailed bibliometric analysis of select musculoskeletal journals, several key findings were noted. First, there was an increase in the number of authors, institutions, countries, references, pages, citations, and corresponding author position overall over time. There were many differences by the geographic region of origin, likely reflecting different socio/cultural attitudes regarding academia and research, as well as the gender composition of the disciplines by geographic region. The overall mentorship of junior female authors has been improving over time by both male and female mentors. There has been an increase in the number of female 1^st^ and corresponding authors, editorial board members, and chief editors, indicating a slow but progressive narrowing of the gender gap, although parity has clearly not yet been achieved. Based on projections, it may take up to a century to achieve gender parity. Therefore, continued active and intentional programs for improvement are needed including continued mentorship, development of orthopaedic opportunities for women in medical school, program development by musculoskeletal societies, and increased representation of women in leadership roles including editorial board and editor-in-chief position.

## Figures and Tables

**Table 1:** Continuous bibliometric variables.

	*n*	Author number	Number of institutions	Number of countries	Number of references	Number of pages	Number of citations	Number of annualized citations	Corresponding author position	Corresponding author position standardized
All-mean ± 1sd (median)		4.7 ± 2.4 (4)	2.0 ± 1.5 (2)	1.2 ± 0.6 (1)	28.6 ± 19.2 (26)	6.7 ± 2.9 (6)	32.0 ± 55.8 (13)	2.98 ± 4.04 (1.9)	2.3 ± 2.3 (1)	0.51 ± 0.34 (0.33)
Journal type										
Clinical	9,641	4.4 ± 2.1	2.0 ± 1.5	1.1 ± 0.5	25.7 ± 17.7	6.1 ± 2.6	31.9 ± 56.5	2.92 ± 4.02	2.0 ± 1.9	0.48 ± 0.33
Basic science	3,178	5.6 ± 3.0	2.3 ± 1.7	1.3 ± 0.8	37.5 ± 20.8	8.3 ± 3.1	32.3 ± 53.6	3.15 ± 4.10	3.3 ± 3.2	0.58 ± 0.38
*p* value	**—**	<10^−15^	<10^−15^	<10^−15^	<10^−15^	<10^−15^	0.000127	0.00000022	<10^−15^	<10^−15^
Specific Journal										
ABME	402	4.5 ± 2.4	2.1 ± 1.3	1.3 ± 0.6	35.9 ± 18.1	12.0 ± 3.8	18.0 ± 26.6	2.47 ± 2.80	2.9 ± 2.4	0.66 ± 0.36
BONE	899	5.8 ± 3.0	2.4 ± 1.7	1.3 ± 0.8	41.4 ± 26.6	7.7 ± 2.5	32.3 ± 54.4	3.40 ± 4.02	3.2 ± 3.1	0.57 ± 0.38
CTI	514	4.9 ± 2.4	2.0 ± 1.4	1.2 ± 0.5	31.7 ± 18.4	6.7 ± 2.8	30.7 ± 63.4	2.06 ± 2.83	2.6 ± 2.7	0.50 ± 0.35
JBMR	746	6.8 ± 3.5	2.7 ± 2.3	1.4 ± 1.0	40.8 ± 18.8	8.6 ± 2.6	43.3 ± 60.0	4.13 ± 5.59	4.1 ± 3.9	0.60 ± 0.40
JOR	617	5.6 ± 2.5	2.3 ± 1.4	1.2 ± 0.5	33.7 ± 14.0	8.0 ± 2.4	29.4 ± 45.2	2.95 ± 3.33	3.2 ± 2.9	0.58 ± 0.38
AJSM	814	4.6 ± 2.0	2.1 ± 1.7	1.2 ± 0.5	32.2 ± 18.4	7.1 ± 2.8	44.8 ± 67.8	5.16 ± 6.15	2.2 ± 2.0	0.48 ± 0.33
Arthroscopy	656	4.3 ± 2.1	1.9 ± 1.2	1.1 ± 0.4	23.9 ± 14.4	6.8 ± 2.8	29.7 ± 45.1	3.14 ± 3.76	2.1 ± 1.9	0.48 ± 0.32
BJJ	893	4.3 ± 2.1	2.0 ± 1.4	1.2 ± 0.5	25.7 ± 18.0	5.5 ± 1.9	36.6 ± 50.1	3.08 +3.45	1.8 ± 1.6	0.43 ± 0.30
FAI	491	4.0 ± 1.8	2.1 ± 1.3	1.1 ± 0.4	25.2 ± 18.4	6.3 ± 2.5	21.7 ± 29.4	2.26 ± 2.11	1.9 ± 1.5	0.51 ± 0.33
Injury	1,105	4.3 ± 2.3	2.0 ± 1.4	1.1 ± 0.4	23.3 ± 20.2	5.2 ± 2.4	11.7 ± 32.0	1.50 ± 2.99	1.7 ± 1.7	0.43 ± 0.30
JAR	877	4.3 ± 1.6	1.8 ± 1.1	1.1 ± 0.4	26.4 ± 14.4	5.2 ± 2.2	17.4 ± 27.6	2.97 ± 3.24	2.0 ± 1.7	0.49 ± 0.34
JBJS	989	4.6 ± 2.3	2.2 ± 1.9	1.2 ± 0.6	30.5 ± 22.1	8.0 ± 2.8	60.7 ± 85.8	4.56 ± 5.28	1.9 ± 2.0	0.43 ± 0.32
JHSA	763	3.7 ± 1.6	1.4 ± 0.8	1.1 ± 0.3	19.7 ± 13.0	6.1 ± 2.3	24.9 ± 33.8	1.77 ± 1.91	2.0 ± 1.6	0.55 ± 0.33
JHSE	450	3.4 ± 1.9	1.4 ± 1.1	1.1 ± 0.5	18.8 ± 14.2	5.3 ± 2.5	20.0 ± 21.3	1.81 ± 2.32	1.5 ± 1.1	0.47 ± 0.29
JOT	444	4.3 ± 1.9	1.8 ± 1.3	1.1 ± 0.3	25.4 ± 15.8	5.8 ± 2.1	20.2 ± 32.3	2.27 ± 2.81	2.2 ± 1.8	0.55 ± 0.34
JPO	593	4.0 ± 1.7	1.9 ± 1.1	1.1 ± 0.3	20.3 ± 10.8	5.0 ± 1.6	16.7 ± 20.3	1.18 ± 1.38	1.9 ± 1.5	0.50 ± 0.31
Spine	1,566	5.1 ± 2.6	2.3 ± 1.7	1.2 ± 0.6	28.3 ± 18.0	6.5 ± 2.5	47.0 ± 79.3	3.46 ± 4.61	2.5 ± 2.6	0.50 ± 0.35
*p* value	**—**	<10^−15^	<10^−15^	<10^−15^	<10^−15^	<10^−15^	<10^−15^	<10^−15^	<10^−15^	<10^−15^
Decade										
1985–1987	1,576	3.0 ± 1.5	1.4 ± 0.7	1.1 ± 0.3	18.3 ± 13.2	5.8 ± 3.3	48.2 ± 75.1	1.55 ± 2.42	1.4 ± 0.9	0.49 ± 0.27
1995–1996	2,830	3.9 ± 1.9	1.7 ± 1.1	1.1 ± 0.4	24.7 ± 17.1	6.0 ± 2.7	51.3 ± 70.3	2.35 ± 3.33	1.7 ± 1.3	0.46 ± 0.30
2005–2006	3,924	4.6 ± 2.1	2.0 ± 1.4	1.2 ± 0.5	29.5 ± 19.1	6.9 ± 2.8	44.7 ± 53.3	4.04 ± 4.85	2.1 ± 2.0	0.48 ± 0.34
2015–2016	4,489	5.8 ± 2.7	2.6 ± 1.9	1.3 ± 0.7	33.9 ± 20.4	7.3 ± 2.8	3.2 ± 4.3	2.95 ± 3.89	3.2 ± 3.0	0.56 ± 0.38
*p* value	**—**	<10^−15^	<10^−15^	<10^−15^	<10^−15^	<10^−15^	<10^−15^	<10^−15^	<10^−15^	2.8^−11^
Geographic Region										
North America	6,651	4.5 ± 2.4	2.0 ± 1.6	1.1 ± 0.5	29.2 ± 19.8	7.0 ± 3.0	36.8 ± 63.0	3.24 ± 4.36	2.5 ± 2.4	0.55 ± 0.35
Asia	1,771	5.4 ± 2.5	2.0 ± 1.3	1.2 ± 0.5	27.4 ± 14.7	6.8 ± 2.9	19.4 ± 33.4	2.32 ± 2.80	2.8 ± 2.8	0.50 ± 0.35
Europe	3,846	4.6 ± 2.4	2.1 ± 1.6	1.3 ± 0.7	28.0 ± 19.7	6.2 ± 2.7	30.4 ± 50.6	2.85 ± 4.03	1.9 ± 2.1	0.43 ± 0.31
Australia/New Zealand	393	4.6 ± 2.2	2.2 ± 1.5	1.2 ± 0.5	31.3 ± 20.2	6.9 ± 3.1	30.1 ± 51.5	3.21 ± 3.56	2.3 ± 2.2	0.53 ± 0.35
Africa	62	3.6 ± 1.9	1.8 ± 1.2	1.2 ± 0.4	18.9 ± 12.4	5.4 ± 2.2	13.3 ± 18.8	1.37 ± 1.54	1.5 ± 1.2	0.43 ± 0.28
Latin America	93	4.8 ± 2.7	1.9 ± 1.2	1.2 ± 0.6	30.4 ± 26.9	6.2 ± 2.7	19.3 ± 30.5	1.96 ± 2.02	1.8 ± 1.9	0.38 ± 0.28
*p* value	**—**	<10^−15^	0.007	<10^−15^	4.2 × 10^−9^	<10^−15^	<10^−15^	<10^−15^	<10^−15^	<10^−15^
Journal Editorial Office Location										
North America	9,857	4.8 ± 2.5	2.1 ± 1.6	1.2 ± 0.6	29.7 ± 19.2	7.0 ± 2.9	34.5 ± 58.4	3.24 ± 4.25	2.5 ± 2.5	0.49 ± 0.34
Europe	2,958	4.3 ± 2.2	1.9 ± 1.4	1.2 ± 0.5	24.8 ± 18.8	5.5 ± 2.4	22.7 ± 44.9	2.12 ± 3.09	1.9 ± 1.8	0.55 ± 0.37
*p* value	**—**	<10^−15^	1.96 × 10^−12^	0.11	<10^−15^	<10^−15^	<10^−15^	<10^−15^	<10^−15^	5.1 × 10^−11^
First Author Gender										
Male	10,191	4.5 ± 2.3	2.0 ± 1.5	1.2 ± 0.5	27.6 ± 18.6	6.6 ± 2.9	33.5 ± 57.3	2.97 ± 4.08	2.2 ± 2.1	0.51 ± 0.34
Female	2,210	5.3 ± 2.8	2.3 ± 1.8	1.2 ± 0.6	33.6 ± 21.6	7.4 ± 2.9	27.9 ± 52.0	3.16 ± 4.02	3.0 ± 3.0	0.48 ± 0.35
*p* value	**—**	<10^−15^	<10^−15^	2 × 10^−7^	<10^−15^	<10^−15^	1.6 × 10^−13^	0.0005	<10^−15^	7.2 × 10^−8^
Corresponding Author Gender										
Male	10,555	4.6 ± 2.4	2.0 ± 1.5	1.2 ± 0.5	27.9 ± 19.0	6.6 ± 2.9	32.5 ± 55.4	2.97 ± 4.09	2.3 ± 2.3	0.54 ± 0.36
Female	1,782	5.2 ± 2.8	2.3 ± 1.8	1.2 ± 0.7	33.4 ± 20.4	7.3 ± 2.9	27.9 ± 50.3	3.14 ± 3.91	2.4 ± 2.7	0.49 ± 0.36
*p* value	**—**	<10^−15^	<10^−15^	0.018	<10^−15^	<10^−15^	2.6 × 10^−9^	0.0017	0.26	3.3 × 10^−8^

ABME = *Annals of Biomedical Engineering*, CTI = *Calcified Tissue International*, JBMR = *Journal of Bone and Mineral Research*, JOR = *Journal of Orthopaedic Research*, AJSM = *American Journal of Sports Medicine*, BJJ = *Bone and Joint Journal*, FAI = *Foot and Ankle International*, JAR = *Journal of Arthroplasty*, JBJS = *Journal of Bone and Joint Surgery*, JHSA = *Journal of Hand Surgery American*, JHSE = *Journal of Hand Surgery European*, JOT = *Journal of Orthopaedic Trauma*, and JPO = *Journal of Pediatric Orthopaedics*.

**Table 2:** Multifactorial ANOVA of continuous variables, with *p* values.

Variable	Author number	Number of institutions	Number of Countries	Number of references	Number of pages	Number of citations	Number of annualized citations	Corresponding author position	Corresponding author position normalized
Decade	8.6 × 10^−12^	9.8 × 10^−12^	1.3 × 10^−11^	1 × 10^−11^	1.1 × 10^−11^	8.9 × 10^−12^	1.1 × 10^−11^	9.6 × 10^−12^	1.5 × 10^−11^
Journal	5.4 × 10^−12^	7.1 × 10^−12^	8.2 × 10^−12^	5.6 × 10^−12^	4.5 × 10^−12^	6.3 × 10^−12^	6.3 × 10^−12^	5.9 × 10^−12^	8.3 × 10^−12^
1^st^ author Gender	0.004	0.09	0.99	0.07	0.002	0.44	0.75	1.9 × 10^−11^	2.1 × 10^−11^
CA gender	0.014	0.75	0.012	0.70	0.72	0.67	0.58	1.9 × 10^−11^	1.9 × 10^−11^
Region	1.5 × 10^−11^	0.005	1.5 × 10^−11^	2.4 × 10^−10^	0.00004	1.1 × 10^−7^	1.6 × 10^−11^	1.4 × 10^−11^	1.1 × 10^−11^
									
Decade	8.7 × 10^−12^	9.8 × 10^−12^	1.3 × 10^−11^	9.9 × 10^−12^	1.1 × 10^−11^	8.9 × 10^−12^	1.8 × 10^−11^	9.6 × 10^−12^	1.5 × 10^−11^
Journal type	1.5 × 10^−11^	2.0 × 10^−11^	1.9 × 10^−11^	1.5 × 10^−11^	1.4 × 10^−11^	0.56	0.007	1.5 × 10^−11^	1.8 × 10^−11^
1^st^ author Gender	0.0002	0.038	0.69	0.04	0.031	0.44	0.82	1.9 × 10^−11^	2.0 × 10^−11^
CA gender	0.09	0.45	0.029	0.37	0.52	0.99	0.93	1.9 × 10^−11^	1.9 × 10^−11^
Region	1.6 × 10^−11^	0.002	1.5 × 10^−11^	1.7 × 10^−11^	1.3 × 10^−11^	1.6 × 10^−11^	1.4 × 10^−11^	1.3 × 10^−11^	1.1 × 10^−11^

**Table 3:** Author gender.

	1^st^ author gender					Corresponding author gender				
	Female	Male	%F	%M	*p* value	Female	Male	%F	%M	*p* value
Journal type										
Clinical	1,180	8,123	12.7	87.3	<10^−15^	978	8,246	10.6	89.4	<10^−15^
Basic science	1,030	2,068	33.2	66.8		804	2,309	25.8	74.2	
Specific journal										
ABME	91	302	23.2	76.8	<10^−15^	71	326	17.9	82.1	<10^−15^
BONE	324	548	37.2	62.8		251	621	28.8	71.2	
CTI	188	316	37.3	62.7		142	365	28.0	72.0	
JBMR	286	445	39.1	60.9		229	505	31.2	68.8	
JOR	141	457	23.6	76.4		111	492	18.4	81.6	
AJSM	107	700	13.3	86.7		96	717	11.8	88.2	
Arthroscopy	85	776	9.9	90.1		41	591	6.5	93.5	
BJJ	86	723	10.6	89.4		76	756	9.1	90.9	
FAI	65	410	13.7	86.3		62	412	13.1	86.9	
Injury	152	900	14.4	85.6		139	923	13.1	86.9	
JAR	85	776	9.9	90.1		84	773	9.8	90.2	
JBJS	110	849	11.5	88.5		91	747	10.9	89.1	
JHSA	106	650	14.0	86.0		73	683	9.7	90.3	
JHSE	56	383	12.8	87.2		38	401	8.7	91.3	
JOT	44	388	10.2	89.8		32	406	7.3	92.7	
JPO	96	489	16.4	83.6		68	515	11.7	88.3	
Spine	223	1,284	14.8	85.2		178	1,322	11.9	88.1	
Decade										
1985–1987	164	1,353	10.8	89.2	<10^−15^	128	1,312	8.9	91.1	<10^−15^
1995–1996	403	2,377	14.5	85.5		329	2,459	11.8	88.2	
2005–2006	619	3,171	16.3	83.7		513	3,291	13.5	86.5	
2015–2016	1,024	3,290	23.7	76.3		812	3,493	18.9	81.1	
Geographic Region										
North America	1,109	5,465	16.9	83.1	<10^−15^	870	5,637	13.4	86.6	2.2 × 10^−14^
Asia	213	1,355	13.6	86.4		169	1,396	10.8	89.2	
Europe	751	2,974	20.2	79.8		634	3,097	17.0	83.0	
Australia/New Zealand	114	267	29.9	70.1		89	293	23.3	76.7	
Journal Editorial Office Location										
North America	1,728	7,869	18.0	82.0	0.33	1,387	8,110	14.6	85.4	0.36
Europe	482	2,322	17.2	82.8		395	2,445	13.9	86.1	
Corresponding author location										
First	1,131	6,182	15.5	84.5	<10^−15^	1,092	6,129	15.1	84.9	0.16
Second	157	806	16.3	83.7		131	841	13.5	86.5	
Other	87	305	22.2	77.8		51	345	12.9	87.1	
Last	782	2,525	23.6	76.4		462	2,883	13.8	86.2	
Single Author										
No	2,157	9,638	97.6	94.9	2.3 × 10^−9^					
Yes	53	552	2.4	5.1						

ABME = *Annals of Biomedical Engineering*, CTI = *Calcified Tissue International*, JBMR = *Journal of Bone and Mineral Research*, JOR = *Journal of Orthopaedic Research*, AJSM = *American Journal of Sports Medicine*, BJJ = *Bone and Joint Journal*, FAI = *Foot and Ankle International*, JAR = *Journal of Arthroplasty*, JBJS = *Journal of Bone and Joint Surgery*, JHSA = *Journal of Hand Surgery American*, JHSE = *Journal of Hand Surgery European*, JOT = *Journal of Orthopaedic Trauma*, and JPO = *Journal of Pediatric Orthopaedics*.

**Table 4:** Author gender combinations.

	FF	FM	MF	MM	%FF	%FM	%MF	%MM	*p* value
All	239	777	367	3,174	5.2	17.1	8.1	69.7	**—**
Journal type									
Clinical	64	393	208	2,376	2.1	12.9	6.8	78.1	<10^−15^
Basic Science	175	384	159	798	11.5	25.3	10.5	52.6	
Specific journal									
ABME	12	44	21	140	5.5	20.3	9.7	64.5	<10^−15^
BONE	51	117	47	193	12.5	28.7	11.5	47.3	
CTI	30	66	20	84	15.0	33.0	10.0	42.0	
JBMR	59	93	37	191	15.5	24.5	9.7	50.3	
JOR	23	64	34	190	7.4	20.6	10.9	61.1	
AJSM	9	32	22	229	3.1	11.0	7.5	78.4	
Arthroscopy	3	19	7	168	1.5	9.6	3.6	85.3	
BJJ	2	25	14	164	1.0	12.2	6.8	80.0	
FAI	5	17	13	135	2.9	10.0	7.6	79.4	
Injury	7	32	22	179	2.9	13.3	9.2	74.6	
JAR	3	30	28	269	0.9	9.1	8.5	81.5	
JBJS	4	26	12	186	1.8	11.4	5.3	81.6	
JHSA	4	26	12	186	1.8	11.4	5.3	81.6	
JHSE	2	18	3	84	1.9	16.8	2.8	78.5	
JOT	3	24	12	152	1.6	12.6	6.3	79.6	
JPO	2	38	19	159	0.9	17.4	8.7	72.9	
Spine	14	84	41	431	2.5	14.7	7.2	75.6	
Decade									
1985–1987	6	46	17	273	1.8	13.5	5.0	79.8	1.4 × 10^−14^
1995–1996	28	106	43	651	3.4	12.8	5.2	78.6	
2005–2006	67	219	118	945	5.0	16.2	8.7	70.1	
2015–2016	138	406	189	1,305	6.8	19.9	9.3	64.0	
Geographic region									
North America	127	455	225	2,016	4.5	16.1	8.0	71.4	1.1 × 10^−7^
Asia	22	88	40	447	3.7	14.7	6.7	74.9	
Europe	69	138	88	604	7.7	15.4	9.8	67.2	
Australia/New Zealand	17	34	10	90	11.3	22.5	6.6	59.6	
Journal editorial office location									
North America	198	636	308	2663	5.2	16.7	8.1	70.0	0.57
Europe	41	141	59	511	5.5	18.8	7.8	68.0	

FF = both authors female, FM = first author female and corresponding author male, MF = first author male and corresponding author female, and MM = both authors male. ABME = *Annals of Biomedical Engineering*, CTI = *Calcified Tissue International*, JBMR = *Journal of Bone and Mineral Research*, JOR = *Journal of Orthopaedic Research*, AJSM = *American Journal of Sports Medicine*, BJJ = *Bone and Joint Journal*, FAI = *Foot and Ankle International*, JAR = *Journal of Arthroplasty*, JBJS = *Journal of Bone and Joint Surgery*, JHSA = *Journal of Hand Surgery American*, JHSE = *Journal of Hand Surgery European*, JOT = *Journal of Orthopaedic Trauma*, and JPO = *Journal of Pediatric Orthopaedics*.

**Table 5:** Changes over time in the number of manuscripts.

	1985–1987	1995–1996	2005–2006	2015–2016	% 1985–1987	% 1995–1996	%2005–2006	% 2015–2016	*p* value
Journal type									
Clinical	1,248	1,974	3,012	3,407	12.9	20.5	31.2	35.3	5.7 × 10^−14^
Basic science	328	856	912	1,082	10.3	26.9	28.7	34.0	
Specific journal									
ABME	35	58	155	154	8.7	14.4	38.6	38.3	<10^−15^
BONE	67	258	219	355	7.5	28.7	24.4	39.5	
CTI	111	187	106	110	21.6	36.4	20.6	21.4	
JBMR	55	237	230	224	7.4	31.8	30.8	30.0	
JOR	60	116	202	239	9.7	18.8	32.7	38.7	
AJSM	86	165	213	350	10.6	20.3	26.2	43.0	
Arthroscopy	33	87	307	229	5.0	13.3	46.8	34.9	
BJJ	159	178	298	258	17.8	19.9	33.4	28.9	
FAI	33	112	188	158	6.7	22.8	38.3	32.2	
Injury	112	188	281	524	10.1	17.0	25.4	47.4	
JAR	37	143	216	481	4.2	16.3	24.6	54.8	
JBJS	154	159	400	276	15.6	16.1	40.4	27.9	
JHSA	179	175	174	235	23.5	22.9	22.8	30.8	
JHSE	75	167	107	101	16.7	37.1	23.8	22.4	
JOT	48	88	125	183	10.8	19.8	28.2	41.2	
JPO	138	137	152	166	23.3	23.1	25.6	28.0	
Spine	154	375	591	446	9.8	23.9	37.7	28.5	
Geographic region									
North America	969	1,560	1,906	2,216	14.6	23.5	28.7	33.3	<10^−15^
Asia	110	263	568	830	6.2	14.9	32.1	46.9	
Europe	451	905	1,270	1,220	11.7	23.5	33.0	31.7	
Australia/New Zealand	31	71	129	162	7.9	18.1	32.8	41.2	
Africa	10	10	16	26	16.1	16.1	25.8	41.9	
Latin America	4	20	35	34	4.3	21.5	37.6	36.6	
Journal editorial office location									
North America	1,119	2,110	3,132	3496	11.4	21.4	31.8	35.5	4.0 × 10^−13^
Europe	457	720	792	993	15.4	24.3	26.7	33.5	
Corresponding author location									
First	1,081	1,823	2,384	2191	14.5	24.4	31.9	29.3	<10^−15^
Second	129	255	334	302	12.6	25.0	32.7	29.6	
Other	38	83	107	193	9.0	19.7	25.4	45.8	
Last	220	539	974	1729	6.4	15.6	28.1	49.9	
Single Author									
No	1,356	2,645	3778	4,418	11.1	21.7	31.0	36.2	<10^−15^
Yes	220	185	146	70	35.4	29.8	23.5	11.3	

ABME = *Annals of Biomedical Engineering*, CTI = *Calcified Tissue International*, JBMR = *Journal of Bone and Mineral Research*, JOR = *Journal of Orthopaedic Research*, AJSM = *American Journal of Sports Medicine*, BJJ = *Bone and Joint Journal*, FAI = *Foot and Ankle International*, JAR = *Journal of Arthroplasty*, JBJS = *Journal of Bone and Joint Surgery*, JHSA = *Journal of Hand Surgery American*, JHSE = *Journal of Hand Surgery European*, JOT = *Journal of Orthopaedic Trauma*, and JPO = *Journal of Pediatric Orthopaedics*.

**Table 6:** Corresponding author location.

Variable	First	Last	% first	% last	*p* value
Journal type					
Clinical	5,977	2,179	73.3	26.7	<10^−15^
Basic science	1,502	1,283	53.9	46.1	
Specific journal					
ABME	172	187	47.9	52.1	<10^−15^
BONE	421	340	55.3	44.7	
CTI	285	155	64.8	35.2	
JBMR	338	346	49.4	50.6	
JOR	286	255	52.9	47.1	
AJSM	515	188	73.3	26.7	
Arthroscopy	427	145	74.7	25.3	
BJJ	638	156	80.4	19.6	
FAI	300	127	70.3	29.7	
Injury	748	170	81.5	18.5	
JAR	488	224	68.5	31.5	
JBJS	621	171	78.4	21.6	
JHSA	621	171	78.4	21.6	
JHSE	638	156	80.4	19.6	
JOT	227	140	61.9	38.1	
JPO	345	131	72.5	27.5	
Spine	880	439	66.7	33.3	
Decade					
1985–1987	1,081	220	83.1	16.9	<10^−15^
1995–1996	1,823	539	77.2	22.8	
2005–2006	2,384	974	71.0	29.0	
2015–2016	2,191	1,729	55.9	44.1	
Geographic region					
North America	3,526	2,129	62.4	37.6	<10^−15^
Asia	938	489	65.7	34.3	
Europe	2,681	704	79.2	20.8	
Australia/New Zealand	221	119	65.0	35.0	
Journal editorial office location					
Europe	2,000	556	78.2	21.8	<10^−15^
North America	5,479	2,906	65.3	34.7	

ABME = *Annals of Biomedical Engineering*, CTI = *Calcified Tissue International*, JBMR = *Journal of Bone and Mineral Research*, JOR = *Journal of Orthopaedic Research*, AJSM = *American Journal of Sports Medicine*, BJJ = *Bone and Joint Journal*, FAI = *Foot and Ankle International*, JAR = *Journal of Arthroplasty*, JBJS = *Journal of Bone and Joint Surgery*, JHSA = *Journal of Hand Surgery American*, JHSE = *Journal of Hand Surgery European*, JOT = *Journal of Orthopaedic Trauma*, and JPO = *Journal of Pediatric Orthopaedics*.

**Table 7:** Single-author manuscripts.

	No	Yes	% no	% yes	*p* value
Journal type					
Clinical	9,108	532	94.5	5.5	6.1 × 10^−10^
Basic science	3,089	89	97.2	2.8	
Specific journal					
ABME	386	16	96.0	4.0	<10^−15^
BONE	865	34	96.2	3.8	
CTI	489	25	95.1	4.9	
JBMR	739	7	99.1	0.9	
JOR	610	7	98.9	1.1	
AJSM	773	41	95.0	5.0	
Arthroscopy	604	52	92.1	7.9	
BJJ	838	55	93.8	6.2	
FAI	470	21	95.7	4.3	
Injury	1,026	79	92.9	7.1	
JAR	833	44	95.0	5.0	
JBJS	940	49	95.0	5.0	
JHSA	711	52	93.2	6.8	
JHSE	406	43	90.4	9.6	
JOT	429	15	96.6	3.4	
JPO	567	26	95.6	4.4	
Spine	1,511	55	96.5	3.5	
Decade					
1985–1987	1,356	220	86.0	14.0	<10^−15^
1995–1996	2,645	185	93.5	6.5	
2005–2006	3,778	146	96.3	3.7	
2015–2016	4,418	70	98.4	1.6	
Geographic region					
North America	6,323	327	95.1	4.9	0.0017
Asia	1,717	54	97.0	3.0	
Europe	3,639	207	94.6	5.4	
Australia/New Zealand	1,717	54	97.0	3.0	
Journal editorial office location					
North America	9,438	419	95.7	4.3	1.1 × 10^−8^
Europe	2759	202	93.2	6.8	

ABME = *Annals of Biomedical Engineering*, CTI = *Calcified Tissue International*, JBMR = *Journal of Bone and Mineral Research*, JOR = *Journal of Orthopaedic Research*, AJSM = *American Journal of Sports Medicine*, BJJ = *Bone and Joint Journal*, FAI = *Foot and Ankle International*, JAR = *Journal of Arthroplasty*, JBJS = *Journal of Bone and Joint Surgery*, JHSA = *Journal of Hand Surgery American*, JHSE = *Journal of Hand Surgery European*, JOT = *Journal of Orthopaedic Trauma*, and JPO = *Journal of Pediatric Orthopaedics*.

**Table 8:** Predictors of a female author from multivariate logistic regression analysis.

By individual journal							
Female first author				Female corresponding author			
Journal	OR	95% CI	*p* value		OR	95% CI	*p* value
CTI	7.9	5.6, 11.2	<10^−15^	JBMR	6.6	4.6, 9.5	<10^−15^
JBMR	7.4	5.3, 10.3	<10^−15^	CTI	6.3	4.3, 9.2	<10^−15^
BONE	6.6	4.8, 9.2	<10^−15^	BONE	5.7	4.0, 8.1	<10^−15^
JOR	3.3	2.4, 4.8	1.1 × 10^−11^	JOR	3.2	2.2, 4.6	4.1 × 10^−9^
ABME	3.2	2.2, 4.7	1.3 × 10^−9^	ABME	3.0	2.9, 4.6	1.7 × 10^−7^
JPO	2.4	1.7, 3.5	0.000002	FAI	2.2	1.4, 3.3	0.0003
Spine	2.1	1.5, 2.9	0.00001	JPO	2.1	1.4, 3.2	0.0004
JHSA	2.0	1.4, 2.9	0.0001	Spine	2.0	1.4, 2.9	0.0001
JHSE	1.8	1.2, 2.7	0.004	Injury	1.8	1.2, 2.6	0.002
FAI	1.8	1.2, 2.7	0.005	AJSM	1.8	1.2, 2.7	0.002
Injury	1.6	1.1, 2.3	0.008	JBJS	1.8	1.2, 2.6	0.004
AJSM	1.6	1.1, 2.2	0.014	JHSA	1.7	1.1, 2.5	0.01
JBJS	1.5	1.1, 2.1	0.024	JAR	1.4	0.9, 2.1	0.09
JOT	1.2	0.8, 1.9	0.40	JHSE	1.4	0.9, 2.3	0.12
BJJ	1.2	0.9, 1.8	0.27	BJJ	1.3	0.9, 2.0	0.17
JAR	1.1	0.7, 1.5	0.76	JOT	1.1	0.7, 1.8	0.68
Arthroscopy	*R*	**—**	**—**	Arthroscopy	*R*	**—**	**—**
Decade				Decade			
2015–2016	2.9	2.4, 3.5	<10^−15^	2015–2016	2.5	2.1, 3.1	<10^−15^
2005–2006	1.7	1.4, 2.1	<10^−15^	2005–2006	1.6	1.3, 2.0	0.000005
1995–1996	1.2	1.0, 1.5	5.7 × 10^−8^	1995–1996	1.2	1.0, 1.5	0.13
1985–1987	*R*	**—**	**—**	1985–1987	*R*	**—**	**—**
Region				Region			
Australia/New Zealand	2.7	2.0, 3.6	3.1 × 10^−12^	Australia/New Zealand	2.4	1.7, 3.2	1.9 × 10^−8^
Europe	1.9	1.6, 2.3	5.5 × 10^−13^	Europe	1.9	1.6, 2.3	1.3 × 10^−11^
North America	1.6	1.3, 1.9	5.6 × 10^−8^	North America	1.5	1.2, 1.8	0.000027
Asia	*R*	**—**	**—**	Asia	*R*	**—**	**—**
By journal type							
Journal type				Journal type			
Basic Science	3.5	3.2, 3.9	<10^−15^	Basic Science	3.0	1.6, 2.3	<10^−15^
Clinical	*R*	**—**	**—**	Clinical	*R*	**—**	**—**
Decade				Decade			
2015–2016	2.6	2.2, 3.1	<10^−15^	2015–2016	2.4	2.0, 3.0	<10^−15^
2005–2006	1.6	1.3, 1.9	0.000002	2005–2006	1.6	1.3, 2.0	0.00001
1995–1996	1.2	1.0, 1.5	0.05	1995–1996	1.2	1.0, 1.5	0.067
1985–1987	*R*	**—**	**—**	1985–1987	*R*	**—**	**—**
Region				Region			
Australia/New Zealand	2.7	2.1, 3.6	8.7 × 10^−13^	Australia/New Zealand	2.4	1.8, 3.3	3.6 × 10^−9^
Europe	1.9	1.6, 1.2	4.9 × 10^−13^	Europe	1.9	1.6, 2.3	4.6 × 10^−12^
North America	1.5	1.3, 1.8	0.000001	North America	1.4	1.2, 1.7	0.00006
Asia	*R*	**—**	**—**	Asia	*R*	**—**	**—**

OR = odds ratio, 95% CI = 95% confidence interval, R = reference value. ABME = *Annals of Biomedical Engineering*, CTI = *Calcified Tissue International*, JBMR = *Journal of Bone and Mineral Research*, JOR = *Journal of Orthopaedic Research*, AJSM = *American Journal of Sports Medicine*, BJJ = *Bone and Joint Journal*, FAI = *Foot and Ankle International*, JAR = *Journal of Arthroplasty*, JBJS = *Journal of Bone and Joint Surgery*, JHSA = *Journal of Hand Surgery American*, JHSE = *Journal of Hand Surgery European*, JOT = *Journal of Orthopaedic Trauma*, and JPO = *Journal of Pediatric Orthopaedics*.

**Table 9:** Predictors of a single author from multivariate logistic regression analysis.

Without author gender				With author gender			
By individual journal							
Journal	OR	95% CI	*p* value	Journal	OR	95% CI	*p* value
Arthroscopy	12.2	5.2, 28.8	1.2 × 10^−8^	Injury	12.8	5.1, 32.2	7.0 × 10^−8^
Injury	11.7	5.0, 27.4	1.4 × 10^−8^	Arthroscopy	12.6	5.0, 32.0	1.2 × 10^−7^
JHSE	10.6	4.4, 25.3	1.3 × 10^−7^	JHSE	11.7	4.5, 30.1	3.8 × 10^−7^
JAR	9.6	4.0, 22.8	2.9 × 10^−7^	JAR	10.1	4.9, 25.9	0.000001
BJJ	7.4	3.1, 17.6	0.000005	BJJ	8.0	3.1, 20.6	0.00001
AJSM	7.0	2.9, 22.8	0.00001	JHSA	7.3	2.9, 18.6	0.00003
JHSA	6.8	2.9, 16.1	0.00001	AJSM	7.2	2.8, 18.6	0.00004
ABME	6.0	2.3, 15.5	0.0002	ABME	6.7	2.4, 18.5	0.0003
JBJS	6.0	2.5, 14.1	0.00005	BONE	6.6	2.6, 17.2	0.0009
BONE	5.5	2.3, 13.4	0.0001	JBJS	6.2	2.4, 15.8	0.0001
FAI	5.3	2.1, 13.4	0.0005	FAI	5.7	2.1, 15.5	0.0007
CTI	4.7	1.9, 11.7	0.0008	CTI	5.3	2.9, 14.0	0.0009
JOT	4.6	1.8, 12.0	0.002	Spine	5.1	2.9, 12.8	0.0006
Spine	4.6	1.8, 12.0	0.004	JOT	4.9	1.8, 13.7	0.002
JPO	4.4	1.8, 10.8	0.0001	JPO	4.7	1.2, 12.4	0.002
JOR	1.5	0.5, 4.6	0.45	JOR	1.7	0.5, 5.4	0.37
JBMR	*R*	**—**	**—**	JBMR	*R*	**—**	**—**
Decade				Decade			
1985–1987	11.0	8.2, 14.6	<10^−15^	1985–1987	10.2	7.6, 13.7	<10^−15^
1995–1996	5.0	3.8, 6.7	<10^−15^	1995–1996	4.8	3.6, 6.5	<10^−15^
2005–2006	2.6	1.9, 3.5	5.8 × 10^−10^	2005–2006	2.5	1.9, 3.4	1.9 × 10^−9^
2015–2016	*R*	**—**	**—**	2015–2016	*R*	**—**	**—**
Region				Region			
North America	1.3	1.0, 1.8	0.066	North America	1.5	1.0, 2.0	0.025
Europe	1.2	0.9, 1.7	0.18	Europe	1.4	0.9, 1.9	0.08
Australia/New Zealand	1.4	0.8, 2.5	0.22	Australia/New Zealand	1.5	0.8, 2.8	0.17
Asia	*R*	**—**	**—**	Asia	*R*	**—**	**—**
				Author Gender			
				Male	1.6	1.2, 2.14	0.003
				Female	*R*	**—**	**—**
By journal type							
Journal type				Journal type			
Clinical	2.0	1.6, 2.5	4.2 × 10^−9^	Clinical	1.9	1.5, 2.4	2.5 × 10^−7^
Basic science	*R*	**—**	**—**	Basic Science	*R*	**—**	**—**
Decade	Decade						
1985–1987	10.2	7.7, 13.5	<10^−15^	1985–1987	9.4	7.1, 12.6	<10^−15^
1995–1996	4.7	3.5, 6.2	<10^−15^	1995–1996	4.5	3.3, 6.0	<10^−15^
2005–2006	2.4	1.8, 3.2	1.2 × 10^−8^	2005–2006	2.3	1.7, 3.1	4.3 × 10^−8^
2015–2016	*R*	**—**	**—**	2015–2016	*R*	**—**	**—**
Region	Region						
Europe	1.4	1.0, 1.9	0.05	North America	1.3	0.9, 1.7	0.14
Australia/New Zealand	1.4	0.8, 2.4	0.27	Europe	1.5	1.1, 2.1	0.016
North America	1.2	0.9, 1.6	0.28	Australia/New Zealand	1.5	0.8, 2.6	0.21
Asia	*R*	**—**	**—**	Asia	*R*	**—**	**—**
				Author gender			
				Male	1.7	1.2, 2.2	0.00087
				Female	*R*	**—**	**—**

OR = odds ratio, 95% CI = 95% confidence interval, R = reference value. ABME = Annals of Biomedical Engineering, CTI = Calcified Tissue International, JBMR = Journal of Bone and Mineral Research, JOR = Journal of Orthopaedic Research, AJSM = American Journal of Sports Medicine, BJJ = Bone and Joint Journal, FAI = Foot and Ankle International, JAR = Journal of Arthroplasty, JBJS = Journal of Bone and Joint Surgery, JHSA = Journal of Hand Surgery American, JHSE = Journal of Hand Surgery European, JOT = Journal of Orthopaedic Trauma, and JPO = Journal of Pediatric Orthopaedics.

**Table 10:** Comparison between author gender and editorial board composition.

	Decade								
	1985–86	1995–96	2005–06	2015–16	% 1985–86	% 1995–96	% 2005–06	% 2015–16	*p* value^
Entire study									
First author									
Female	164	403	619	1,024	10.8	14.5	16.3	23.7	<10^15^
Male	1,353	2,377	3,171	3,290	89.2	85.5	83.7	76.3	
Corresponding author									
Female	128	329	513	812	8.9	11.8	13.5	18.9	<10^15^
Male	1,312	2,459	3,291	3,493	91.1	88.2	86.5	81.1	
Editorial board									
Female	26	78	100	144	4.4	7.8	8.6	11.2	0.000001
Male	567	925	1,068	1,144	95.6	92.2	91.4	88.8	
By Journal Type									
Clinical									
First author									
Female	95	177	331	577	7.9	9.1	11.4	17.8	<10^15^
Male	1,107	1,759	2,584	2,673	92.1	90.9	88.6	82.2	
Corresponding author									
Female	78	145	279	476	7.0	7.5	9.6	14.7	<10^15^
Male	1,041	1,795	2,641	2,769	93.0	92.5	90.4	85.3	
Editorial board									
Female	11	33	48	39	2.9	4.7	5.8	4.5	0.27
Male	374	675	778	826	97.1	95.3	94.2	95.5	
Basic science									
First Author									
Female	69	226	288	447	21.9	26.8	32.9	42.2	<10^15^
Male	246	618	587	612	78.1	73.2	67.1	57.8	
Corresponding author									
Female	50	184	234	336	15.6	21.7	26.5	31.7	2.8 × 10^−11^
Male	271	664	650	724	84.4	78.3	73.5	68.3	
Editorial board									
Female	15	45	52	105	7.2	15.3	15.2	24.8	4.7 × 10^−8^
Male	193	250	290	318	92.8	84.7	84.8	75.2	
By geographic region									
North America									
Corresponding author									
Female	66	175	261	368	7.4	11.3	13.8	16.9	5.3 × 10^−14^
Male	827	1,374	1,626	1,810	92.6	88.7	86.2	83.1	
Editorial board									
Female	16	50	60	97	4.0	8.3	8.5	11.0	0.00008
Male	387	554	648	785	96.0	91.7	91.5	89.0	
Europe									
Corresponding author									
Female	53	121	187	273	12.9	13.7	15.1	22.8	4.9 × 10^−9^
Male	359	761	1,055	922	87.1	86.3	84.9	77.2	
Editorial board									
Female	9	15	19	36	6.7	7.2	8.7	14.1	0.0046
Male	126	192	200	219	93.3	92.8	91.3	85.9	
Asia									
Corresponding author									
Female	6	18	44	101	6.4	7.0	8.8	14.2	0.00015
Male	88	240	458	610	93.6	93.0	91.2	85.8	
Editorial board									
Female	0	1	2	5	0.0	1.7	3.4	5.1	0.16
Male	18	59	56	93	100.0	98.3	96.6	94.9	
Australia/New Zealand									
Corresponding author									
Female	3	10	16	60	10.3	14.3	13.1	37.3	0.000004
Male	26	60	106	101	89.7	85.7	86.9	62.7	
Editorial board									
Female	0	0	1	3	0.0	0.0	8.3	11.1	0.096
Male	8	19	11	24	100.0	100.0	91.7	88.9	
Latin America									
Corresponding author									
Female	0	3	3	7	0.0	15.0	8.6	20.6	0.27
Male	4	17	32	27	100.0	85.0	91.4	79.4	
Editorial board									
Female	0	0	0	0	0.0	0.0	0.0	0.0	**—**
Male	4	7	5	9	100.0	100.0	100.0	100.0	
Africa									
Corresponding author									
Female	0	2	2	3	0.0	22.2	12.5	11.5	0.74
Male	8	7	14	23	100.0	77.8	87.5	88.5	
Editorial board									
Female	1	0	0	1	20.0	0.0	0.0	11.1	0.81
Male	4	5	1	8	80.0	100.0	100.0	88.9	
By location of journal editorial office									
North America									
First author									
Female	107	302	498	821	9.7	14.5	16.4	24.4	<10^15^
Male	997	1,784	2,545	2,543	90.3	85.5	83.6	75.6	
Corresponding author									
Female	74	252	423	638	7.2	12.1	13.9	19.0	<10^15^
Male	947	1,833	2,614	2,716	92.8	87.9	86.1	81.0	
Editorial board									
Female	22	64	86	118	4.7	7.8	8.6	11.2	0.000023
Male	448	757	919	933	95.3	92.2	91.4	88.8	
Europe									
First author									
Female	57	101	121	203	13.8	14.6	16.2	21.4	0.000053
Male	356	593	626	747	86.2	85.4	83.8	78.6	
Corresponding author									
Female	54	77	90	174	12.9	11.0	11.7	18.3	
Male	365	626	677	777	87.1	89.0	88.3	81.7	0.00029
Editorial board									
Female	4	14	14	26	3.3	7.7	8.6	11.0	
Male	119	168	149	211	96.7	92.3	91.4	89.0	0.014

^ = Cochran linear trend test.

## Data Availability

The data are available from the corresponding author upon request.

## Supplementary Materials

Supplementary Materials S1 Appendix: Continuous bibliometric variables over time for each journal. S2 Appendix: Analyses by US regions. S3 Appendix: Analyses by Canadian regions. S4 Appendix: Analyses by European regions. S5 Appendix: Analyses by the four major Asian countries.
