# Supplementary material for: Bibliometric Analysis of the English Musculoskeletal Literature over the Last 30 Years
Source: ScientificWorldJournal. 2021 Apr 27;2021:5548481. doi: 10.1155/2021/5548481 (PMC8096573; doi:10.1155/2021/5548481)
Supplement: Supplementary Materials — S1 Appendix: Continuous bibliometric variables over time for each journal. S2 Appendix: Analyses by US regions. S3 Appendix: Analyses by Canadian regions. S4 Appendix: Analyses by European regions. S5 Appendix: Analyses by the four major Asian countries. [file 5548481.f1.zip › 5548481.f1/S1 appendix.doc]

**S1 Appendix: Continuous bibliometric variables over time for each journal**

|  | **Number of authors** | | | | | **Number of Institutions** | | | | | **Number of countries** | | | | | **Number of References** | | | | | **Number of pages** | | | | | **Corresponding author position standardized** | | | | | | **Corresponding author position** | | | | | **Number of normalized citations** | | | | | |
| --- | --- | --- | --- | --- | --- | --- | --- | --- | --- | --- | --- | --- | --- | --- | --- | --- | --- | --- | --- | --- | --- | --- | --- | --- | --- | --- | --- | --- | --- | --- | --- | --- | --- | --- | --- | --- | --- | --- | --- | --- | --- | --- |
| **Journal** | **1985-87** | **1995-1996** | **2005-2006** | **2015-2016** | **% change** | **1985-87** | **1995-1996** | **2005-2006** | **2015-2016** | **% change** | **1985-87** | **1995-1996** | **2005-2006** | **2015-2016** | **% change** | **1985-87** | **1995-1996** | **2005-2006** | **2015-2016** | **% change** | **1985-87** | **1995-1996** | **2005-2006** | **2015-2016** | **% change** | **Journal** | **1985-87** | **1995-1996** | **2005-2006** | **2015-2016** | **% change** | **1985-87** | **1995-1996** | **2005-2006** | **2015-2016** | **% change** | **1985-87** | **1995-1996** | **2005-2006** | **2015-2016** | **% change** | **% change^** |
| **ABME** | 3.14 | 3.38 | 3.93 | 5.76 | 83.3 | 1.43 | 1.59 | 1.99 | 2.55 | 78.2 | 1.01 | 1.05 | 1.15 | 1.25 | 23.4 | 21.66 | 33.78 | 33.65 | 42.23 | 95.0 | 15.49 | 12.16 | 10.90 | 12.34 | -20.3 | ABME | 0.62 | 0.68 | 0.68 | 0.69 | 11.9 | 1.41 | 1.49 | 1.81 | 2.89 | 105.7 | 2.47 | 3.44 | 6.83 | 5.61 | 127.4 | 176.9 |
| **BONE** | 3.55 | 4.39 | 5.88 | 7.08 | 99.3 | 1.49 | 1.81 | 2.41 | 2.91 | 95.1 | 1.14 | 1.12 | 1.19 | 1.48 | 29.5 | 23.87 | 32.22 | 39.72 | 52.36 | 119.4 | 5.76 | 6.37 | 8.85 | 8.39 | 45.6 | BONE | 0.46 | 0.53 | 0.62 | 0.64 | 38.1 | 1.63 | 2.14 | 2.56 | 3.81 | 134.0 | 0.59 | 1.49 | 2.52 | 3.20 | 445.3 | 329.5 |
| **CTI** | 3.29 | 4.47 | 5.48 | 6.46 | 96.6 | 1.47 | 1.63 | 2.30 | 2.94 | 100.0 | 1.00 | 1.22 | 1.11 | 1.14 | 13.7 | 23.34 | 27.97 | 39.65 | 38.90 | 66.6 | 5.72 | 5.02 | 7.92 | 9.50 | 66.1 | CTI | 0.76 | 0.50 | 0.58 | 0.61 | -19.6 | 1.43 | 1.75 | 1.59 | 2.39 | 66.8 | 0.97 | 1.82 | 2.98 | 3.46 | 257.1 | 207.6 |
| **JBMR** | 4.07 | 5.40 | 7.10 | 8.52 | 109.3 | 1.42 | 2.13 | 2.82 | 3.43 | 142.1 | 1.06 | 1.05 | 1.08 | 1.18 | 11.6 | 30.09 | 37.00 | 41.97 | 46.41 | 54.2 | 7.55 | 7.35 | 8.91 | 9.99 | 32.4 | JBMR | 0.57 | 0.52 | 0.58 | 0.73 | 27.4 | 1.09 | 1.41 | 1.69 | 2.97 | 171.8 | 1.76 | 2.26 | 3.34 | 3.41 | 93.2 | 89.5 |
| **JOR** | 3.68 | 4.52 | 5.18 | 6.86 | 86.2 | 1.35 | 1.79 | 1.81 | 3.10 | 130.0 | 1.18 | 1.19 | 1.37 | 1.46 | 23.5 | 25.20 | 32.08 | 33.06 | 37.28 | 47.9 | 8.58 | 8.21 | 7.37 | 8.25 | -3.9 | JOR | 0.44 | 0.51 | 0.59 | 0.65 | 47.5 | 1.37 | 2.00 | 3.56 | 4.28 | 211.6 | 0.88 | 2.76 | 4.57 | 3.62 | 312.2 | 420.1 |
| **AJSM** | 2.94 | 3.38 | 3.99 | 5.87 | 99.4 | 1.27 | 1.58 | 2.14 | 2.53 | 99.7 | 1.11 | 1.17 | 1.29 | 1.36 | 23.1 | 18.72 | 24.90 | 34.46 | 37.53 | 100.5 | 5.36 | 5.66 | 7.61 | 7.96 | 48.5 | AJSM | 0.54 | 0.52 | 0.47 | 0.51 | -6.4 | 1.85 | 2.03 | 3.12 | 3.95 | 113.6 | 1.63 | 1.69 | 2.79 | 2.43 | 48.5 | 70.6 |
| **ARTHROSCOPY** | 2.30 | 3.67 | 3.75 | 5.68 | 146.7 | 1.36 | 1.70 | 1.59 | 2.37 | 73.9 | 1.06 | 1.06 | 1.13 | 1.20 | 12.8 | 13.33 | 19.63 | 20.35 | 31.94 | 139.6 | 6.15 | 6.18 | 6.02 | 8.22 | 33.7 | ARTHROSCOPY | 0.63 | 0.46 | 0.52 | 0.54 | -14.9 | 1.94 | 1.59 | 1.74 | 1.74 | -10.2 | 1.06 | 1.71 | 2.47 | 2.65 | 150.3 | 133.1 |
| **BJJ** | 2.65 | 3.73 | 4.45 | 5.45 | 105.8 | 1.53 | 1.71 | 2.07 | 2.53 | 64.9 | 1.01 | 1.05 | 1.10 | 1.23 | 22.0 | 14.74 | 20.01 | 28.18 | 33.40 | 126.6 | 4.57 | 4.93 | 5.32 | 6.54 | 43.2 | BJJ | 0.63 | 0.46 | 0.41 | 0.44 | -30.8 | 1.29 | 1.31 | 1.64 | 2.03 | 56.7 | 0.37 | 0.74 | 2.58 | 1.43 | 290.5 | 604.8 |
| **FAI** | 2.64 | 3.40 | 3.77 | 5.09 | 93.0 | 1.55 | 1.69 | 1.82 | 2.74 | 77.3 | 1.07 | 1.15 | 1.13 | 1.35 | 25.8 | 23.39 | 19.97 | 24.68 | 29.78 | 27.3 | 8.94 | 5.47 | 5.71 | 7.11 | -20.5 | FAI | 0.76 | 0.54 | 0.51 | 0.48 | -36.4 | 1.54 | 1.24 | 1.94 | 2.47 | 60.2 | 3.62 | 4.72 | 5.93 | 3.00 | -17.2 | 63.8 |
| **INJURY** | 2.50 | 2.99 | 3.70 | 5.48 | 119.1 | 1.17 | 1.41 | 1.69 | 2.45 | 109.7 | 1.09 | 1.13 | 1.20 | 1.26 | 15.1 | 9.64 | 10.79 | 25.62 | 29.36 | 204.5 | 3.34 | 3.14 | 6.19 | 5.76 | 72.5 | INJURY | 0.61 | 0.52 | 0.50 | 0.40 | -34.0 | 1.43 | 1.43 | 1.61 | 2.33 | 63.3 | 1.50 | 2.93 | 3.85 | 3.25 | 116.8 | 157.3 |
| **JAR** | 3.19 | 3.46 | 3.94 | 4.88 | 53.0 | 1.19 | 1.22 | 1.59 | 2.11 | 77.8 | 1.07 | 1.24 | 1.51 | 1.59 | 48.2 | 18.35 | 22.57 | 22.93 | 29.67 | 61.7 | 7.05 | 5.86 | 6.06 | 4.56 | -35.4 | JAR | 0.52 | 0.57 | 0.50 | 0.51 | -2.5 | 2.29 | 2.55 | 3.90 | 6.25 | 173.0 | 1.00 | 1.91 | 7.00 | 4.29 | 328.8 | 599.4 |
| **JBJS** | 3.40 | 3.87 | 4.60 | 5.70 | 67.9 | 1.54 | 2.11 | 1.95 | 3.01 | 95.6 | 1.04 | 1.06 | 1.09 | 1.09 | 3.9 | 23.20 | 28.92 | 31.54 | 34.03 | 46.7 | 7.75 | 8.08 | 8.18 | 7.70 | -0.7 | JBJS | 0.59 | 0.38 | 0.47 | 0.47 | -21.6 | 1.28 | 1.57 | 2.02 | 2.75 | 114.6 | 1.05 | 1.67 | 2.38 | 1.95 | 85.2 | 125.3 |
| **JHSA** | 2.70 | 3.16 | 3.98 | 4.68 | 73.3 | 1.27 | 1.30 | 1.59 | 1.41 | 11.4 | 1.05 | 1.01 | 1.07 | 1.35 | 27.8 | 13.78 | 17.91 | 22.63 | 23.50 | 70.6 | 5.20 | 5.56 | 6.55 | 6.81 | 31.0 | JHSA | 0.57 | 0.57 | 0.55 | 0.61 | 7.7 | 1.17 | 1.32 | 1.51 | 2.01 | 71.3 | 0.62 | 1.05 | 1.95 | 3.81 | 515.3 | 214.3 |
| **JHSE** | 2.32 | 3.27 | 3.67 | 4.32 | 86.1 | 1.11 | 1.08 | 1.17 | 2.27 | 104.9 | 1.05 | 1.11 | 1.17 | 1.28 | 21.9 | 13.75 | 16.30 | 18.00 | 27.80 | 102.2 | 4.01 | 4.83 | 4.94 | 7.31 | 82.1 | JHSE | 0.62 | 0.48 | 0.48 | 0.54 | -12.9 | 1.38 | 2.12 | 3.00 | 4.40 | 217.9 | 1.44 | 2.65 | 3.77 | 2.77 | 93.0 | 162.1 |
| **JOT** | 3.19 | 3.58 | 4.15 | 5.04 | 58.2 | 1.44 | 1.61 | 1.68 | 2.11 | 47.1 | 1.02 | 1.08 | 1.10 | 1.13 | 10.3 | 19.08 | 21.18 | 28.66 | 26.82 | 40.5 | 6.98 | 5.84 | 5.51 | 5.70 | -18.3 | JOT | 0.51 | 0.53 | 0.50 | 0.63 | 22.5 | 1.42 | 1.69 | 1.89 | 2.97 | 109.8 | 1.44 | 2.65 | 3.77 | 2.77 | 93.0 | 162.1 |
| **JPO** | 3.07 | 3.16 | 4.55 | 4.78 | 55.7 | 1.39 | 1.50 | 2.24 | 2.18 | 56.7 | 1.02 | 1.09 | 1.11 | 1.11 | 9.1 | 15.67 | 20.13 | 21.53 | 23.02 | 47.0 | 4.36 | 5.09 | 4.93 | 5.56 | 27.7 | JPO | 0.53 | 0.54 | 0.44 | 0.56 | 6.6 | 1.39 | 1.47 | 1.87 | 2.60 | 87.0 | 0.61 | 0.90 | 2.07 | 1.08 | 78.5 | 239.9 |
| **SPINE** | 3.29 | 4.06 | 5.13 | 6.70 | 103.8 | 1.40 | 1.92 | 2.09 | 3.07 | 119.0 | 1.08 | 1.14 | 1.19 | 1.33 | 23.6 | 19.40 | 26.17 | 30.43 | 30.42 | 56.8 | 4.85 | 6.38 | 6.37 | 7.49 | 54.3 | SPINE | 0.49 | 0.41 | 0.42 | 0.74 | 50.0 | 1.27 | 1.40 | 1.91 | 4.77 | 274.7 | 2.84 | 3.75 | 4.56 | 1.97 | -30.7 | 60.6 |

ABME = Annals of Biomedical Engineering, CTI = Calcified Tissue International, JBMR = Journal of Bone and Mineral Research, JOR = Journal of Orthopaedic Research, AJSM = American Journal of Sports Medicine, BJJ = Bone and Joint Journal, FAI = Foot and Ankle International, JAR = Journal of Arthroplasty, JBJS = Journal of Bone and Joint Surgery, JHSA = Journal of Hand Surgery American, JHSE = Journal of Hand Surgery European, JOT = Journal of Orthopaedic Trauma, JPO = Journal of Pediatric Orthopaedics.

The % change compares 2015-1016 to 1985-1987, except for ^, which compares 2005-2006 to 1985-1987
