# Supplementary material for: Bibliometric Analysis of the English Musculoskeletal Literature over the Last 30 Years
Source: ScientificWorldJournal. 2021 Apr 27;2021:5548481. doi: 10.1155/2021/5548481 (PMC8096573; doi:10.1155/2021/5548481)
Supplement: Supplementary Materials — S1 Appendix: Continuous bibliometric variables over time for each journal. S2 Appendix: Analyses by US regions. S3 Appendix: Analyses by Canadian regions. S4 Appendix: Analyses by European regions. S5 Appendix: Analyses by the four major Asian countries. [file 5548481.f1.zip › 5548481.f1/S2 appendix.doc]

**Supplemental 2 Appendix: Analyses by US regions**

The regions comprise the following states as defined by the US Census Bureau:

**1) Northeast:** Connecticut, Maine, Massachusetts, New Hampshire, New Jersey, New York, Pennsylvania, Rhode Island, Vermont **2)** **South:** Alabama, Arkansas, Delaware, District of Columbia, Florida, Georgia, Kentucky, Louisiana, Maryland, Mississippi, North Carolina, Oklahoma, South Carolina, Tennessee, Texas, Virginia, West Virginia **3) Midwest:** Illinois, Indiana, Iowa, Kansas, Michigan, Minnesota, Missouri, Nebraska, North Dakota, Ohio, South Dakota, Wisconsin **4) West:** Alaska, Arizona, California, Colorado, Hawaii, Idaho, Montana, Nevada, New Mexico, Oregon, Utah, Washington, Wyoming

**Table**

|  | **Midwest** | **% Midwest** | **North East** | **% North East** | **South** | **% South** | **West** | **% West** | **p value** |
| --- | --- | --- | --- | --- | --- | --- | --- | --- | --- |
| **All** | 1644 | 26.9 | 1737 | 28.4 | 1534 | 25.1 | 1199 | 19.6 | - |
| **All %** |  |  |  |  |  |  |  |  |  |
| **Decade** |  |  |  |  |  |  |  |  |  |
| **1985-1987** | 236 | 14.4 | 252 | 14.5 | 252 | 16.4 | 164 | 13.7 | 0.002 |
| **1995-1996** | 375 | 22.8 | 357 | 20.6 | 376 | 24.5 | 320 | 26.7 |  |
| **2005-2006** | 471 | 28.6 | 502 | 28.9 | 436 | 28.4 | 339 | 28.3 |  |
| **2015-2016** | 562 | 34.2 | 626 | 36.0 | 470 | 30.6 | 376 | 31.4 |  |
| **Journal Type** |  |  |  |  |  |  |  |  |  |
| **Basic Science** | 389 | 23.7 | 457 | 26.3 | 317 | 20.7 | 317 | 26.4 | 0.0004 |
| **Clinical** | 1255 | 76.3 | 1280 | 27.6 | 1217 | 26.3 | 882 | 73.6 |  |
| **Single Author** |  |  |  |  |  |  |  |  |  |
| **No** | 1572 | 95.6 | 1656 | 95.3 | 1448 | 94.4 | 1126 | 93.9 | 0.13 |
| **Yes** | 72 | 4.4 | 81 | 4.7 | 86 | 5.6 | 73 | 6.1 |  |
| **First Author Gender** | |  |  |  |  |  |  |  |  |
| **Female** | 227 | 13.9 | 325 | 19.0 | 236 | 15.5 | 186 | 15.7 | 0.0008 |
| **Male** | 1401 | 86.1 | 1386 | 81.0 | 1283 | 84.5 | 1001 | 84.3 |  |
| **Corresponding Author Gender** | | |  |  |  |  |  |  |  |
| **Female** | 203 | 12.7 | 225 | 13.5 | 165 | 11.0 | 155 | 13.2 | 0.013 |
| **Male** | 1401 | 87.3 | 1445 | 86.5 | 1339 | 89.0 | 1020 | 86.8 |  |
| **Gender Combination** | |  |  |  |  |  |  |  |  |
| **FF** | 31 | 4.3 | 39 | 3.9 | 17 | 2.2 | 20 | 3.4 | 0.033 |
| **FM** | 88 | 12.3 | 133 | 13.3 | 109 | 13.9 | 64 | 10.8 |  |
| **MF** | 65 | 9.1 | 59 | 5.9 | 39 | 5.0 | 41 | 6.9 |  |
| **MM** | 529 | 74.2 | 772 | 77.0 | 619 | 79.0 | 467 | 78.9 |  |

**Graphs**

By decade (p = 0.0022).

By journal type (p = 0.0004).

By first author gender (p = 0.0008).

 By corresponding author gender (p = 0.013).

By author gender combination (p = 0.033).
