# Supplementary material for: Bibliometric Analysis of the English Musculoskeletal Literature over the Last 30 Years
Source: ScientificWorldJournal. 2021 Apr 27;2021:5548481. doi: 10.1155/2021/5548481 (PMC8096573; doi:10.1155/2021/5548481)
Supplement: Supplementary Materials — S1 Appendix: Continuous bibliometric variables over time for each journal. S2 Appendix: Analyses by US regions. S3 Appendix: Analyses by Canadian regions. S4 Appendix: Analyses by European regions. S5 Appendix: Analyses by the four major Asian countries. [file 5548481.f1.zip › 5548481.f1/S3 appendix.doc]

**Supplemental 3 Appendix: Analyses by Canadian regions**

Canada was arbitrarily divided into three regions consisting of the 1) East (Quebec, Labrador, New Brunswick, Newfoundland, Nova Scotia, Prince Edward Island); 2) Midwest (Ontario, Manitoba, Saskatchewan); and 3) West (British Columbia, Alberta).

**Table**

|  | **East** | **% East** | **Midwest** | **% Midwest** | **West** | **% West** | **p value** |
| --- | --- | --- | --- | --- | --- | --- | --- |
| **All** | 100 | 19.6 | 354 | 69.5 | 55 | 10.8 | - |
| **Decade** |  |  |  |  |  |  |  |
| **1985-1987** | 13 | 13.0 | 47 | 13.3 | 5 | 9.1 | 0.041 |
| **1995-1996** | 21 | 21.0 | 103 | 29.1 | 6 | 10.9 |  |
| **2005-2006** | 28 | 28.0 | 100 | 28.2 | 22 | 40.0 |  |
| **2015-2016** | 38 | 38.0 | 104 | 29.4 | 22 | 40.0 |  |
| **Journal Type** |  |  |  |  |  |  |  |
| **Basic Science** | 44 | 44.0 | 97 | 27.4 | 17 | 30.9 | 0.0066 |
| **Clinical** | 55 | 56.0 | 257 | 72.6 | 38 | 69.1 |  |
| **Single Author** |  |  |  |  |  |  |  |
| **No** | 93 | 96.0 | 343 | 96.9 | 54 | 98.2 | 0.72 |
| **Yes** | 4 | 4.0 | 10 | 2.8 | 1 | 1.8 |  |
| **First Author Gender** |  |  |  |  |  |  |  |
| **Female** | 26 | 26.5 | 90 | 25.9 | 14 | 25.5 | 0.99 |
| **Male** | 72 | 73.5 | 258 | 74.1 | 40 | 72.7 |  |
| **Corresponding Author Gender** | |  |  |  |  |  |  |
| **Female** | 23 | 24.0 | 57 | 15.3 | 12 | 22.2 | 0.18 |
| **Male** | 73 | 76.0 | 292 | 83.7 | 43 | 79.6 |  |
| **Gender Combination** |  |  |  |  |  |  |  |
| **FF** | 7 | 15.6 | 8 | 4.7 | 3 | 10.7 | 0.005 |
| **FM** | 11 | 24.4 | 41 | 24.1 | 6 | 21.4 |  |
| **MF** | 8 | 17.8 | 8 | 4.7 | 4 | 14.3 |  |
| **MM** | 19 | 42.2 | 113 | 66.5 | 15 | 53.6 |  |

**Graphs**

By decade (p = 0.041).

By journal type (p = 0.0066).

By gender combination (p = 0.005).
