# Supplementary material for: Bibliometric Analysis of the English Musculoskeletal Literature over the Last 30 Years
Source: ScientificWorldJournal. 2021 Apr 27;2021:5548481. doi: 10.1155/2021/5548481 (PMC8096573; doi:10.1155/2021/5548481)
Supplement: Supplementary Materials — S1 Appendix: Continuous bibliometric variables over time for each journal. S2 Appendix: Analyses by US regions. S3 Appendix: Analyses by Canadian regions. S4 Appendix: Analyses by European regions. S5 Appendix: Analyses by the four major Asian countries. [file 5548481.f1.zip › 5548481.f1/S4 appendix.doc]

**Supplemental 4 Appendix: Analyses by European regions**

**Geographic Division**

Europe was arbitrarily dividend into three regions consisting of the 1) British Isles (United Kingdom, Northern Ireland), 2) Nordic (Denmark, Sweden, Norway, Finland), and 3) Continental (all other countries).

**Table**

|  | **British Isles** | **% British Isles** | **Continental** | **% Continental** | **Nordic** | **% Nordic** | **p value** |
| --- | --- | --- | --- | --- | --- | --- | --- |
| **All** | 1419 | 36.9 | 1865 | 48.5 | 562 | 14.6 | - |
| **Decade** |  |  |  |  |  |  |  |
| **1985-1987** | 239 | 16.8 | 115 | 6.2 | 97 | 17.3 | < 10^15^ |
| **1995-1996** | 371 | 26.1 | 370 | 19.8 | 164 | 29.2 |  |
| **2005-2006** | 447 | 31.5 | 666 | 35.7 | 157 | 27.9 |  |
| **2015-2016** | 362 | 25.5 | 714 | 28.3 | 144 | 25.6 |  |
| **Journal Type** |  |  |  |  |  |  |  |
| **Basic Science** | 208 | 14.7 | 545 | 29.2 | 184 | 32.7 | < 10^15^ |
| **Clinical** | 1211 | 85.3 | 1320 | 70.8 | 378 | 67.3 |  |
| **Single Author** |  |  |  |  |  |  |  |
| **No** | 1306 | 92.0 | 1809 | 97.0 | 524 | 93.2 | 1.0 x 10^-9^ |
| **Yes** | 113 | 8.0 | 56 | 3.0 | 38 | 6.8 |  |
| **First Author Gender** |  |  |  |  |  |  |  |
| **Female** | 223 | 16.7 | 388 | 21.2 | 140 | 25.3 | 0.00004 |
| **Male** | 1115 | 83.3 | 1445 | 78.8 | 414 | 74.7 |  |
| **Corresponding Author Gender** |  |  |  |  |  |  |  |
| **Female** | 178 | 13.1 | 328 | 17.9 | 128 | 23.5 | 1.2 x 10^-7^ |
| **Male** | 1178 | 86.7 | 1503 | 82.1 | 416 | 76.5 |  |
| **Gender Combination** |  |  |  |  |  |  |  |
| **FF** | 11 | 3.1 | 53 | 10.3 | 5 | 5.7 | 0.0008 |
| **FM** | 67 | 19.1 | 110 | 21.4 | 16 | 18.2 |  |
| **MF** | 27 | 7.7 | 54 | 10.5 | 7 | 8.0 |  |
| **MM** | 246 | 70.1 | 298 | 57.9 | 60 | 68.2 |  |

**Graphs**

By decade (p < 10^-15^).

By journal type (p < 10^-15^).

By author gender combination (p = 0.0008).

By single author (p = 1 x 10^-9^).

By first author gender (p = 0.00004).

By corresponding author gender (p = 1.2 x 10^-7^).

**By former Eastern Bloc status**

**Table**

|  | **Former Eastern Bloc Status** | | | | |
| --- | --- | --- | --- | --- | --- |
|  | **Yes** | **% Yes** | **No** | **% No** | **p value** |
| **All** | 106 | 2.8 | 3740 | 97.2 | - |
| **Decade** |  |  |  |  |  |
| **1985-1987** | 8 | 7.5 | 443 | 11.8 | .013^ |
| **1995-1996** | 19 | 17.9 | 886 | 23.7 |  |
| **2005-2006** | 35 | 33.0 | 1235 | 33.0 |  |
| **2015-2016** | 44 | 41.5 | 1176 | 31.4 |  |
| **Journal Type** |  |  |  |  |  |
| **Basic Science** | 19 | 17.9 | 918 | 24.5 | 0.14 |
| **Clinical** | 87 | 82.1 | 2822 | 75.5 |  |
| **Single Author** |  |  |  |  |  |
| **No** | 96 | 90.6 | 3543 | 94.7 | .076 |
| **Yes** | 10 | 9.4 | 197 | 5.3 |  |
| **First Author Gender** |  |  |  |  |  |
| **Female** | 21 | 20.0 | 730 | 20.2 | 1.00 |
| **Male** | 84 | 80.0 | 2890 | 79.8 |  |
| **Corresponding Author Gender** |  |  |  |  |  |
| **Female** | 18 | 17.5 | 616 | 17.0 | 0.89 |
| **Male** | 85 | 82.5 | 3012 | 83.0 |  |
| **Gender Combination** |  |  |  |  |  |
| **FF** | 2 | 9.1 | 67 | 7.2 | 0.86 |
| **FM** | 4 | 18.2 | 189 | 20.3 |  |
| **MF** | 1 | 4.5 | 87 | 9.3 |  |
| **MM** | 15 | 68.2 | 589 | 63.2 |  |

^ Cochran linear trend test
