# Supplementary material for: Bibliometric Analysis of the English Musculoskeletal Literature over the Last 30 Years
Source: ScientificWorldJournal. 2021 Apr 27;2021:5548481. doi: 10.1155/2021/5548481 (PMC8096573; doi:10.1155/2021/5548481)
Supplement: Supplementary Materials — S1 Appendix: Continuous bibliometric variables over time for each journal. S2 Appendix: Analyses by US regions. S3 Appendix: Analyses by Canadian regions. S4 Appendix: Analyses by European regions. S5 Appendix: Analyses by the four major Asian countries. [file 5548481.f1.zip › 5548481.f1/S5 appendix.doc]

**Supplemental 5 Appendix: Analyses by the four major Asian countries**

**Table**

|  | **China** | **% China** | **Israel** | **% Israel** | **Japan** | **% Japan** | **Korea** | **% Korea** | **p value** |
| --- | --- | --- | --- | --- | --- | --- | --- | --- | --- |
| **All** | 334 | 23.3 | 124 | 8.6 | 702 |  | 276 |  | - |
| **All %** | 23.3 |  | 8.6 |  | 48.9 |  | 19.2 |  | - |
| **Decade** |  |  |  |  |  |  |  |  |  |
| **1985-1987** | 3 | 0.9 | 29 | 23.4 | 53 | 7.5 | 0 | 0.0 | < 10^-15^ |
| **1995-1996** | 16 | 4.8 | 31 | 25.0 | 172 | 24.5 | 7 | 2.5 |  |
| **2005-2006** | 63 | 18.9 | 34 | 27.4 | 255 | 36.3 | 92 | 33.3 |  |
| **2015-2016** | 252 | 75.4 | 30 | 24.2 | 222 | 31.6 | 177 | 64.1 |  |
| **Journal Type** |  |  |  |  |  |  |  |  |  |
| **Basic Science** | 87 | 26.0 | 41 | 33.1 | 223 | 31.8 | 36 | 13.0 | 3 x 10^-8^ |
| **Clinical** | 247 | 74.0 | 83 | 66.9 | 479 | 68.2 | 240 | 87.0 |  |
| **Single Author** |  |  |  |  |  |  |  |  |  |
| **No** | 326 | 97.6 | 120 | 96.8 | 689 | 98.1 | 271 | 98.2 | 0.75 |
| **Yes** | 8 | 2.4 | 4 | 3.2 | 13 | 1.9 | 5 | 1.8 |  |
| **First Author Gender** | | | | | | | | | |
| **Female** | 55 | 21.5 | 24 | 18.2 | 56 | 8.3 | 35 | 15.0 | 1.3 x 10^-7^ |
| **Male** | 201 | 78.5 | 108 | 81.8 | 618 | 91.7 | 198 | 85.0 |  |
| **Corresponding Author Gender** | | | | | | | | | |
| **Female** | 55 | 21.5 | 13 | 10.7 | 40 | 6.1 | 25 | 11.2 | 4.7 x 10^-10^ |
| **Male** | 201 | 78.5 | 108 | 89.3 | 618 | 93.9 | 198 | 88.8 |  |
| **Gender Combination** | | | | | | | | | |
| **FF** | 8 | 5.9 | 1 | 2.3 | 4 | 2.2 | 5 | 3.8 | 7.3 x 10^-8^ |
| **FM** | 27 | 19.9 | 11 | 25.0 | 18 | 9.9 | 17 | 13.0 |  |
| **MF** | 22 | 16.2 | 0 | 0.0 | 2 | 1.1 | 8 | 6.1 |  |
| **MM** | 79 | 58.1 | 32 | 72.7 | 157 | 86.7 | 101 | 77.1 |  |
| **%MM** | 58.1 |  | 72.7 |  | 86.7 |  | 77.1 |  |  |

**Graphs**

By decade (p < 10^-15^).

By journal type (p = 3 x 10^-8^).

By first author gender (p = 1.3 x 10^-7^)

By corresponding author gender (p = 4.7 x 10^-10^).

By author gender combinations (p = 7.3 x 10^-8^).
